# Distinguishing patients with idiopathic epilepsy from solitary cysticercus granuloma epilepsy and biochemical phenotype assessment using a serum biomolecule profiling platform

**DOI:** 10.1371/journal.pone.0237064

**Published:** 2020-08-21

**Authors:** Jay S. Hanas, James Randolph Sanders Hocker, Betcy Evangeline, Vasudevan Prabhakaran, Anna Oommen, Vedantam Rajshekhar, Douglas A. Drevets, Hélène Carabin

**Affiliations:** 1 Department of Biochemistry, University of Oklahoma Health Sciences Center, Oklahoma City, OK, United States of America; 2 Department of Neurological Sciences, Christian Medical College, Vellore, India; 3 Department of Internal Medicine, University of Oklahoma Health Sciences Center, and the Veterans Administration Medical Center, Oklahoma City, OK, United States of America; 4 Department of Biostatistics and Epidemiology, University of Oklahoma Health Sciences Center, Oklahoma City, OK, United States of America; 5 Department of Pathology and Microbiology, Faculty of Veterinary Medicine, Université de Montréal, Saint-Hyacinthe, Canada; Fondazione IRCCS Istituto Neurologico Carlo Besta, ITALY

## Abstract

A major source of epilepsy is Neurocysticercosis (NCC), caused by *Taenia solium* infection. Solitary cysticercus granuloma (SCG), a sub-group of NCC induced epilepsy, is the most common form of NCC in India. Current diagnostic criteria for SCG epilepsy require brain imaging which may not be available in communities where the disease is endemic. Identification of serum changes and potential biomolecules that could distinguish SCG epilepsy from idiopathic generalized epilepsy (IE), without the initial need for imaging, could assist in disease identification, understanding, and treatment. The objective here was to investigate, using mass spectrometry (MS), sera biomolecule differences between patients with SCG epilepsy or IE to help distinguish these disorders based on physiological differences, to understand underlying phenotypes and mechanisms, and to lay ground work for future therapeutic and biomarker analyses. Sera were obtained from patients with SCG or IE (N = 29 each group). Serum mass peak profiling was performed with electrospray ionization (ESI) MS, and mass peak area means in the two groups were compared using leave one [serum sample] out cross validation (LOOCV). Serum LOOCV analysis identified significant differences between SCG and IE patient groups (*p* = 10^−20^), which became non-significant (*p* = 0.074) when the samples were randomly allocated to the groups and reanalyzed. Tandem MS/MS peptide analysis of serum mass peaks from SCG or IE patients was performed to help identify potential peptide/protein biochemical and phenotypic changes involving these two forms of epilepsy. Bioinformatic analysis of these peptide/protein changes suggested neurological, inflammatory, seizure, blood brain barrier, cognition, ion channel, cell death, and behavior related biochemical systems were being altered in these disease states. This study provides groundwork for aiding in distinguishing SCG and IE patients in minimally invasive, lower-cost manners, for improving understanding of underlying epilepsy mechanisms, and for further identifying discriminatory biomarkers and potential therapeutic targets.

## Introduction

Epilepsy is the most common neurological disorder affecting approximately 1% of the world’s population [1, 2]. Its prevalence is two to three times higher in lower income countries where mortality is significantly higher than in higher income regions [2–4]. In approximately 60% of those individuals affected, epileptogenesis is initiated by known structural causes including stroke, traumatic brain injury, and infection [5]. Majority of the remainder 40% of epilepsy cases are caused by unknown genetic and environmental sources termed idiopathic epilepsy (IE), and a small percentage of epilepsies are caused by known gene mutations which reveal themselves in infancy [6]. Neurocysticercosis (NCC) is an infection of the central nervous system caused by the larvae of the tapeworm *Taenia solium*, accounting for approximately 30% of epilepsy cases in endemic areas, and is the major cause of epilepsy world-wide [3, 7–11]. Although basic mechanisms involved in the pathogenesis of epilepsy from *T*. *solium* are not well understood, inflammatory responses appear common in NCC-related epilepsy as well as idiopathic generalized epilepsy (IE) [1, 12]. In India, solitary cysticercus granuloma (SCG), a single stable or degenerating intraparenchymal cysticercus cyst [7–10], is the most common form of NCC. This contrasts with other endemic regions where multiple NCC cysts (MNCC) in the brain are more common [3, 7]. Diagnosis of SCG, from other seizure disorders, in patients presenting with seizures requires brain imaging with contrast-enhanced computerized tomography (CT) or magnetic resonance imaging (MRI), and may not be accessible or affordable to a large number of individuals in endemic areas. Accurate diagnosis is important because most patients with SCG will require treatment with an anti-parasitic and an anti-inflammatory drugs which is different from IE patients where no structural or metabolic cause, such as a tumor, for epilepsy can be identified.

Previous work suggests mass spectrometry (MS) analysis of peripheral blood could aid in NCC diagnosis [13]. This is important because current serological tests are unreliable in diagnosing NCC and sub-groups like SCG in the absence of brain imaging. The serological enzyme-linked immune-electrotransfer blot (EITB) has high sensitivity (proportion of test positives among those truly positive) in detecting patients with multiple NCC lesions, but a much lower sensitivity in patients with SCG (about 50 to 60%) [14]. As reported earlier, an all-liquid MS platform approach using unfractionated serum was able to distinguish NCC patients from IE patients with high sensitivity and specificity in well-characterized patient groups [13]. However, a direct comparison between SCG and IE has not been demonstrated previously. A hypothesis in the present study is that SCG and IE will elicit disease-specific and systemic host responses reflected in the peripheral blood that can be measured and distinguished by serum mass peak profiling using electro-spray ionization mass spectrometry (ESI-MS). In the present study, the extent to which ESI-MS could distinguish patients with SCG from those with IE was tested, based only on their respective serum biomolecule mass peak profiles. In addition, biochemical phenotypes and cellular pathways possibly at play in these disorders, as reflected from the tandem MS/MS structural analysis of peptides and proteins in sera, were also examined to help understand underlying mechanisms and provide physiological basis for the SCG versus IE patient discriminations.

## Materials and methods

### Study participant descriptions

This cross-sectional study was conducted among patients with active epilepsy aged 18 to 51 years seeking care at the Department of Neurological Sciences, Christian Medical College (CMC) and Hospital, Vellore, India between January 2013 and October 2014. The study was approved by the Institutional Review Boards of CMC Vellore and of the University of Oklahoma Health Sciences Center, Oklahoma, USA. Written informed consents were obtained prior to recruitment and specimen retrieval. Study participants consisted of patients diagnosed with SCG-associated epilepsy or patients with IE. The IE patients had no evidence of NCC or other structural brain lesions on CT (N = 8)/MRI (N = 21) performed with contrast and were also seronegative for antigens and antibodies to the larval stages of *T*. *solium*. All patients had had at least one seizure in the previous 7 months and were required to be free of anti-inflammatory drugs for at least 7 days prior to collection of blood sample. Only patients aged between 18 and 51 years old were eligible. The maximum age was chosen to reduce possible confounding effects due to the presence and treatment of co-existing chronic medical conditions. Patients who were tested by the treating physician for HIV, HCV and HBV as part of routine care and found positive were excluded, while patients testing negative or not tested were eligible. All enrolled patients underwent plain or contrast enhanced CT or MRI of the brain. All images were read by one of the authors (VR), and the lesions seen in the images of SCG patients were categorized using previously described recommendations [9, 15]. In the SCG group, diagnosis was made on contrast MRI in 23 patients and on a contrast CT brain-scan in 6 patients. IE was defined according to the operational definition of the International League Against Epilepsy [10]. Serum from patients with SCG (n = 29) and IE (n = 29) underwent ESI-MS leave one out (serum sample) cross validation (LOOCV) analysis [13]. From these, 10 age and sex-matched patient samples from the SCG or IE groups were selected for MS/MS (ESI-tandem MS) analysis. The socio-demographic and clinical characteristics of the patient population are summarized in Table 1.

**Table 1:** Socio-demographic and clinical characteristics of eligible study groups.

Variable	Category	Study Group patients: N (% of patients)	
		SCG ^a^	IE ^b^
**Sample size**		29	29
**Gender**	Male	24 (83)	19 (66)
**Age**	Mean (SD)	30.4 (10.3)	26.9 (7.2)
**Cigarette smoking**	Yes	4 (14)	4 (14)
**Betel leaf / Arecaareca nut chewing**	Yes	4 (14)	3 (10)
**Time since last seizure**	1–3 months	25 (86)	21 (72)
	3–7 months	4 (14)	8 (28)
**No of lifetime seizures**	1	9 (31)	0 (0)
	2–5	16 (55)	9 (31)
	6–10	1 (3)	2 (7)
	>10	3 (11)	18 (62)
**Type of last seizure**	Generalized	20 (69)	24 (83)
	Partial	6 (21)	1 (3)
	Partial with secondary generalization	3 (10)	4 (14)

N = number of patients in group

^a^ SCG: Solitary Cysticercus Granuloma (Neurocysticercosis)

^b^ IE: Idiopathic generalized epilepsy[13]

### Serum sample collection and measurement of exposure and current infection status with *T*. *solium* cysticercosis

Sera were obtained at the CMC Hospital, Vellore according to standard procedures [16]. Sera aliquots (250 μl) were frozen at -80 ^o^C, and not reused after initial freezing and thawing. All sera were tested with serum EITB for cysticercal antibodies and Ag ELISA for cysticercal antigens using monoclonal antibodies to excretory/secretory products of *T*. *saginata* metacestodes [15–17].

### Electrospray mass spectrometry of sera from SCG and IE patients

An LCQ ADVANTAGE ion-trap electrospray MS instrument (ThermoFisher), was used for LOOCV analysis of serum MS spectra and for tandem MS/MS peptide/protein structural identifications. Full-range calibration of the LCQ was performed following recommended manufacturer protocols. The spectral data were analyzed as described previously [13]. Briefly, three mass spectra were obtained from each serum sample over an m/z (mass divided by charge) range of 400 to 2000. Spectral data was extracted using the manufacturer's software (Qual Browser: version 1.4SR1), normalized to a sum value of 100 intensity units in non-overlapping segments of 10 m/z. MS spectral peak assignments and areas were calculated as centroid m/z peak area values (valley to valley) using Mariner Data Explorer 4.0.0.1 software (Applied BioSystems). The same serum samples (SCG and IE) were also analyzed on a lower resolution compact desk-top single quadrupole ESI-MS instrument (Expression CMS, Advion, Inc., Ithaca, NY) as described previously [13].

For MS/MS mass peak peptide/protein structure identifications, 108 parental unit-Dalton m/z ions encompassing the m/z range of 900 to 1008 were analyzed as detailed previously [18]. This m/z range was chosen based on empirically-determined optimal machine performance for MS/MS analysis of unfractionated serum samples. A 35% fragmentation ionization energy was used for each peak, and each parent ion m/z was isolated, fragmented, and observed for 5 minutes. Analysis of MS/MS signals was performed using ThermoFisher Proteome Discoverer 1.0 SP1 on human and *T*. *solium* non-redundant databases downloaded from National Center for Biotechnology Information (NCBI). Serum samples contained on average 1.95 (range: 0–5) parent ions with significant differences of standard MS spectral data between the pre and post MS/MS scans of the 108 parental ions observed. MS/MS search-related settings were as follows: [enzyme name = no-enzyme (no digest)], precursor mass tolerance = 1.8 Da, fragment mass tolerance = 0.8 Da, b & y ions were scored, and dynamic modifications were noted for oxidation (C, M amino acids), phosphorylation (S, T, Y), methylation (C), all with maximum of 4 modifications per peptide. Protein identifications required a minimum of two unique peptides and a cross correlation range (Xcorr) ≥1.7, in line with previous studies [18–20]. Identified sequences were searched using Basic Local Alignment Search Tool (BLAST) analysis. A “hit” in the protein database search is scored for a MS/MS scan when the Xcorr, identifying a peptide sequence, is higher than or equal to a 1.7 cut off. Each serum sample was scanned multiple times for a total of 5 minutes duration at each m/z. Each identification of a peptide or protein sequence from the database was termed a “hit”, and the number of patient sera samples indicating the presence of the same peptide/protein are also reported. Identified protein names and the number of Identified MS/MS sequence “hits” were imported each as log_2_ ratios of SCG/IE for Ingenuity Pathway Analysis (IPA, QIAGEN) [21]. MS/MS “hits”, sera numbers, and IPA use of “hits” further described in S11 File, Remarks Concerning Methods. Proteins were manually inspected and verified for protein function using Medline/PubMed.

### Statistical and quantitative analysis

**Fig 1:**
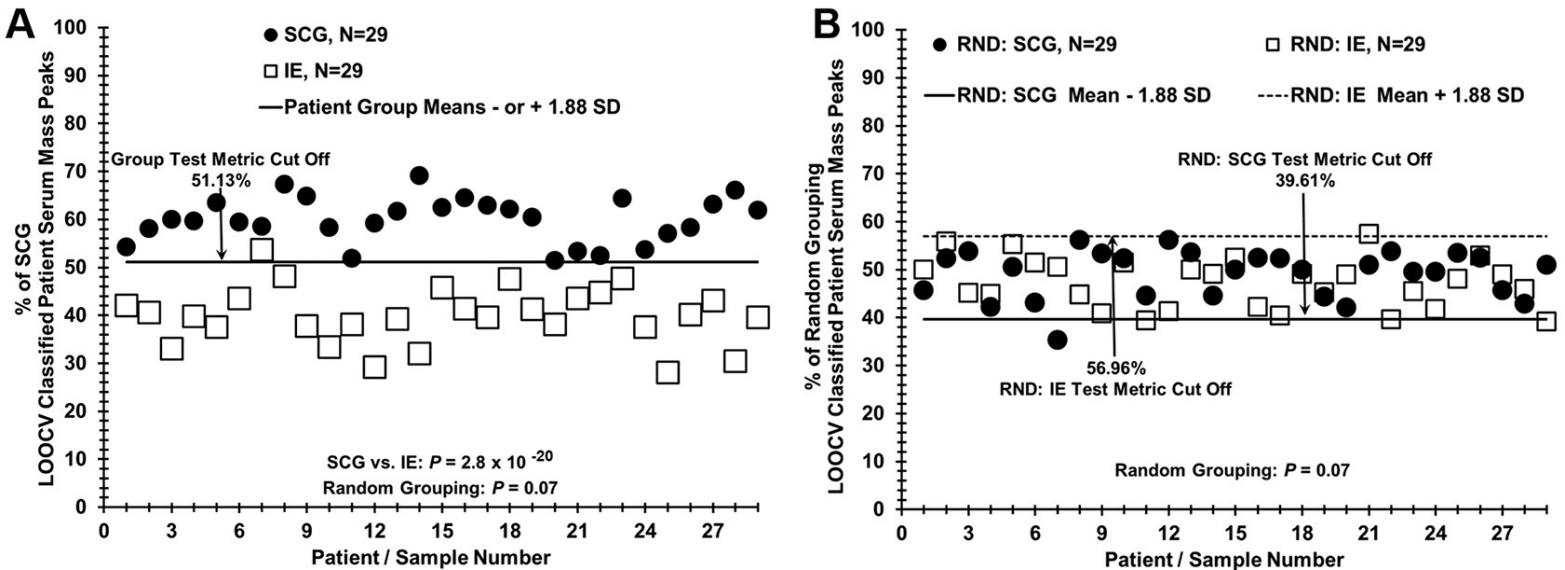
LOOCV mass peak analysis for distinguishing sera from patients with SCG vs patients with IE. (A) Patient scores from the % LOOCV classified mass peak dataset were used to compare SCG (filled circles) and IE (open squares) patients. The “Group Test Metric Cut Off” represents the combined cut off values of both SCG and IE groups and is set at equidistant differences of 1.88 SD’s from each respective group mean (–1.88SD higher scoring group) or (+ 1.88SD lower scoring group). An individual patient serum score > “Cut Off” value suggests higher similarity to the SCG group. Whereas, a score ≤ “Cut Off” value suggests higher similarity to the IE group. (B) Random grouping of patients demonstrates a lack of separation between patient groups. Specific group cut off lines are shown and now represent different values, due to random patient grouping, even though the calculation methodology did not change.

Analysis of mass spectral peaks followed methods described before [13]. Briefly, a leave one [serum sample] out cross validation (LOOCV) method was used to distinguish serum samples of SCG patients from serum samples of IE patients S1 Fig panel A [22–24]. Triplicate averaged peak values were compared between the SCG and IE patients using one-tailed Student’s *t* -tests assuming unequal variance, alternatively leaving out one sample (one sample from the SCG or IE group) to build a series of unique N– 1 LOOCV “left in” significant mass peak datasets. The mass peaks of each “left out” sample were then compared to all the “left in” mass peaks in their unique N-1 LOOCV dataset. This comparison involves the use of a peak classification value (PCV) used to classify each “left out” sample at each significant “left in” peak of each N-1 LOOCV dataset. Whether a “left out” peak area falls above or below the PCV determines if it should be classified into the IE or SCG group. For example, peak 965 in S1B Fig is classified as a “SCG” peak in the “left in” database. If the 965 peak from the “left out” sample has a peak area above the PCV then it is classified as a “SCG” peak. If it falls below or equal to this PCV then the “left out” peak is classified as an “IE” peak. Use of the PCV is further explained in S11 File, Remarks Concerning Methods. Peak classifications are performed for all “left out” peaks in all “left out” serum samples against their respective N-1 “left in” LOOCV mass peak databases resulting in a summed sera score (patient score). Notably, this procedure results in sera samples displaying a combination of “SCG peaks” and “IE peaks”. The % of total mass peaks classified as SCG for the left-in dataset is assigned each “left out” sample and plotted on the y axis versus the individual serum samples on the x-axis as in Fig 1.

To check for over-fitting of such large datasets, each sample was randomized to either the SCG or IE group using the RND (randomization) function in Excel and manually balanced to retain gender and age ratios of the initial groups [13, 22–24]. Upon randomization, the identical LOOCV mass peak analysis was performed again exactly as described above. The Cohen’s *d* values were calculated from the % LOOCV means and standard deviations to get a sense of the effect size observed when comparing two groups [25]. Cohen’s *d* serves as an indirect measure of statistical power to detect the strength of a difference between two groups when one is present. Statistical power using given sample sizes is estimated as described [25, 26].

### Test metrics

**Fig 2:**
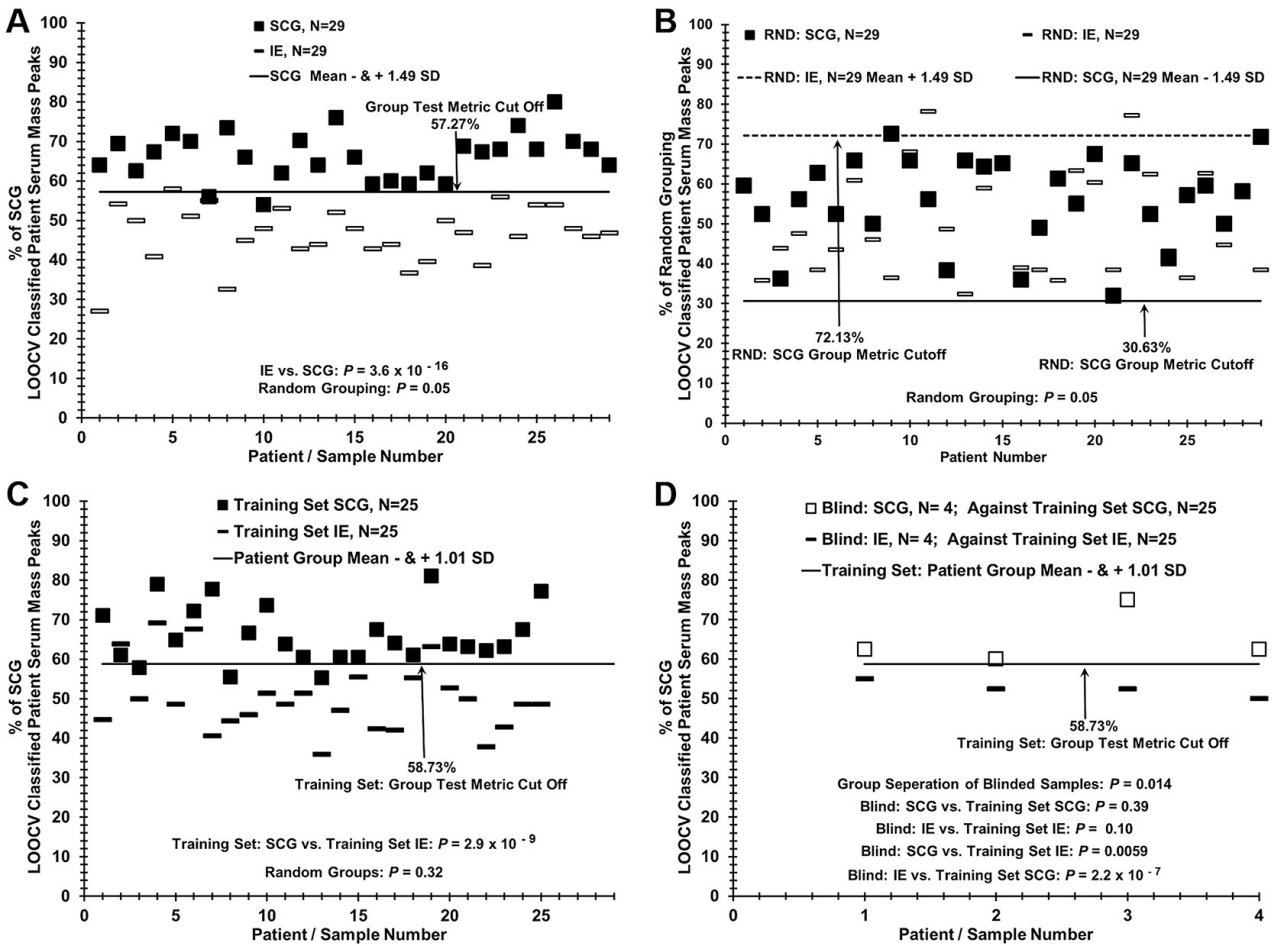
Analysis of blinded “left out” sera sample set using a training set comparison of patients with SCG versus patients with IE. (A) Six blinded SCG and IE samples from each group were removed from the database and the remaining samples (n = 23 each group) were used as a training set. A % LOOCV classified mass peak group test metric cut off of 47.4% was established 1.8 SD’s from each respective group mean as described in the methods. (B) Random grouping of patients demonstrates a lack of separation between patient groups. (C) Six fully blinded samples each from SCG and IE groups were tested against the test metric cut off of 47.4% determined by the training group dataset. (D) ROC representation was based on the classification of each patient verses its known group membership. The random (area under the curve, AUC = 0.50) ROC area (solid diagonal line) indicates theoretical true random group membership. True groupings of data in Fig 2 panel A (AUC = 0.98) and random grouping in Fig 2 panel B (AUC = 0.64) are shown.

**Fig 3:**
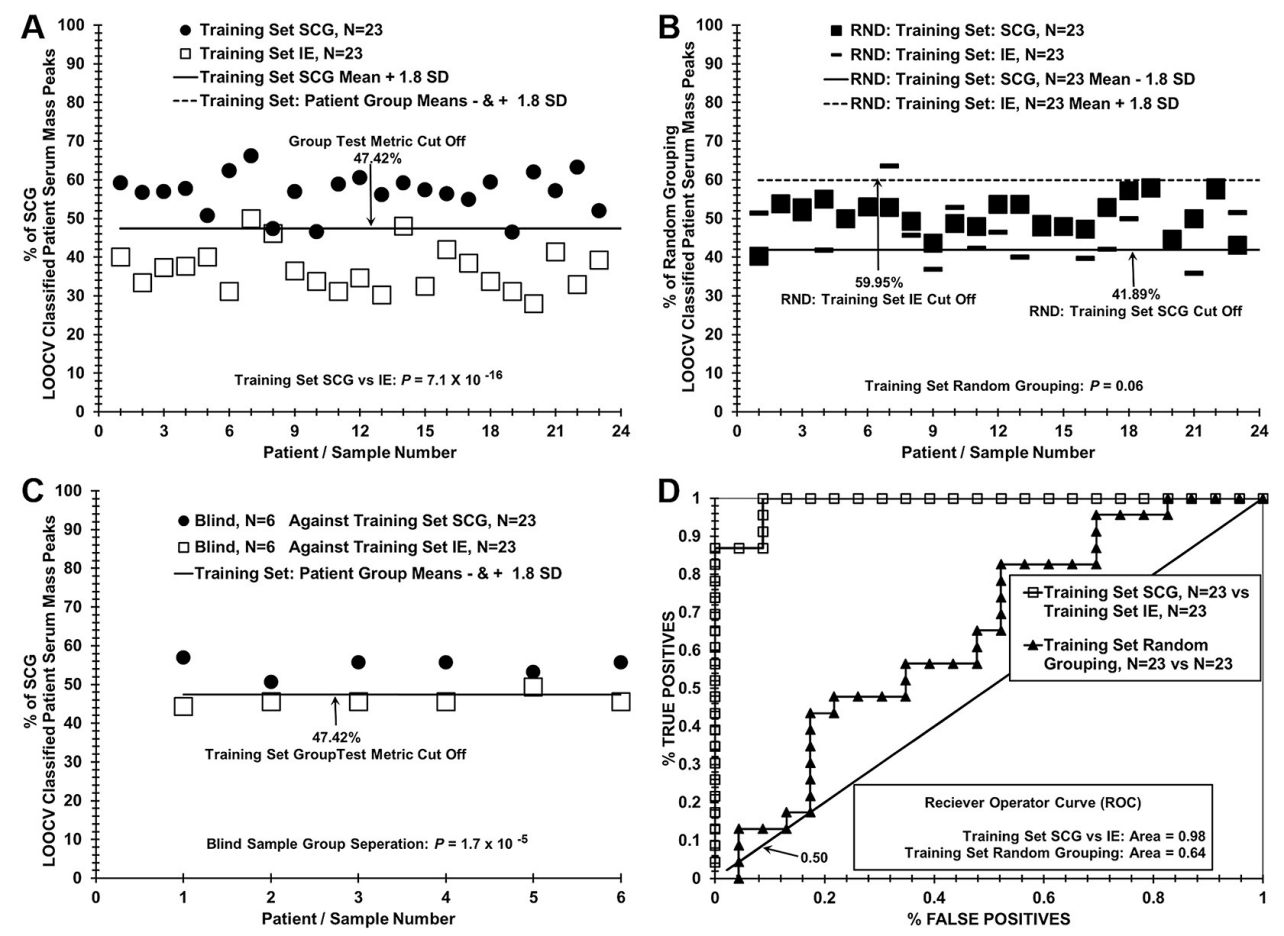
A small footprint desk-top ESI-MS demonstrates is able to determine group membership of SCG or IE patient samples. Advion MS analysis (small footprint MS) of SCG and IE patients. The lower ion sensitivity and limited m/z (50–1200) range of the instrument is still demonstrated as effective for proper classification of both SCG and IE patients. Panel A Patient scores for the LOOCV dataset (N = 29 each group) used for the blinded analysis of six SCG and six IE patients is presented with group separations between patient with SCG (filled squares) vs IE (open rectangles). The “Group Test Metric Cut Off” represents the combined cut off values of both the SCG and IE group membership and is set at equidistant differences of 1.49 standard deviations from each respective group mean ± (–, higher scoring group) or (+, lower scoring group) the respective group standard deviation and are represented by an equivalent value. (B) Random grouping of patients demonstrates a lack of group separation. (C) Patient scores from a “Training” dataset used to test fully blinded samples are displayed. (D) Patient scores resulting from testing of five SCG and five IE individuals are presented after scoring against a fixed value training database resulting in 8 of 8 correct classification of patient samples when compared to the “Group Test Metric Cut Off” in panel C.

For each comparison, a group test metric “cut off” value was calculated from the mean % LOOCV classified peaks from each group minus (SCG), or plus (IE), an equivalent number of SD’s as previously described [13]. LOOCV cut off values were used to determine True Positive (TP), False Positives (FP), True Negative (TN), and False Negative (FN) values for classifying SCG and IE patients into the proper group (Figs 1–3). The sensitivity was defined as the percentage of SCG patients classified as SCG because their % of total LOOCV was above the SCG LOOCV threshold cut off. The specificity was defined as the percentage of IE patients classified as IE because their % of total LOOCV was at or below the SCG LOOCV threshold cut off. Receiver operator characteristic (ROC) curve analysis was performed using each patients % LOOCV classified peaks as described previously [13, 27–29].

## Results

### Distinguishing patients with SCG epilepsy versus IE epilepsy using ESI-MS

The ability of ESI-MS to distinguish sera from patients with SCG from those with IE is illustrated in Fig 1A. The results show a clear demarcation between SCG patients and IE patients in the % of SCG LOOCV classified patient serum mass peaks. The calculated cut-off value of 51.13% SCG LOOCV classified mass peaks minus 1.88 times SD yielded a strong separation between groups evidenced by the very low *p*-value (4.74x10^-20^). In contrast, when data from study subjects are randomized nearly all samples are classified as members of both the SCG and IE groups (Fig 1B). Indeed, all samples below the dashed line are classified as members of the RND IE group while all samples above the solid line are classified as members of the RND SCG group. The lack of clear difference between the randomized groups is evidenced by a much larger *p*-value found when the mean total % LOOCV are compared (p = 0.074). These results are consistent with minimal over-fitting and support the presence of a physiological basis for the differences between the SCG and IE study groups.

### Classification of blinded SCG and IE patient sera against a known training set

A blinded sample experiment was performed to by removing six SCG samples and six IE samples from the dataset used in Fig 1A, and the LOOCV analysis was re-run using the remaining 23 SCG and 23 IE samples for a “training set” (Fig 2 panel A). The training set distinguished samples from the two study groups with a *p* value of 7.1 x10^-16^, and randomization of training set samples resulted in a *p* value of 0.06, suggesting minimal over fitting (Fig 2B). The 12 “blinded” samples were then classified using the SCG LOOCV test metric cut off determined by the training set in Fig 2 panel A and displayed in Fig 2 panel C. In this comparison 11 out of 12 samples were correctly classified however one blind sample from the IE patient group was above the cut-off value of 47.4% of SCG training set, so would have mistakenly been classified as SCG. Fig 2 panel D shows the two ROC curves generated from both the training dataset and the random training set. The training dataset showed maximal sensitivity and specificity at a value of 0.98 for the true pathology dataset and 0.64 for the random grouping training set.

### Distinguishing patients with SCG epilepsy versus IE epilepsy with a lower-resolution desk-top ESI-MS instrument

The ability of a lower-cost ESI-MS instrument with a different mass analyzer to distinguish SCG and IE sera samples is exhibited in Fig 3. The results (panel A) show a demarcation between SCG patients and IE patients in the % of SCG LOOCV classified patient serum mass peaks, with a *p*-value for this group discrimination of 4.74 10^−20^, and a cut-off value (for discerning false negatives and false positives) of 57.27%.

At least two false negative samples are apparent in this group discrimination (darkened squares). When serum samples from study subjects are randomized between the two groups and their mass peaks are re-analyzed by the LOOCV process, nearly all samples are classified as members of both the SCG and IE groups (Fig 3B), although the group discriminatory *p* value is at the 0.05 significant level. These results are consistent with minimal over-fitting and support the presence of a physiological basis for the differences between the SCG and IE study groups, even using the lower-resolution MS instrument. In Fig 3, panel C and D, a similar blinded sample experiment was performed with the lower resolution instrument to that in Fig 2. Eight sera samples (4 SCG and 4 IE) were removed from the training set in panel C and analyzed by the LOOCV process in panel D. All 8 were identified correctly. When this number was increased to 10 or 12, the results were worse (not shown) than the higher resolution instrument in Fig 1.

### Test metric data for SCG and IE serum profiling comparisons

Table 2 summarizes the test metrics for the binary group discriminatory LOOCV data in Figs 1–3. The pathological groups tested in binary fashion from these figures are listed in the far-left column. The performance of ESI-MS to classify subjects into their true study group was high, with a sensitivity and a specificity of 100% and 97%, respectively, when the full LOOCV dataset was used. When the smaller training set was used, the ESI-MS still performed well with sensitivity and specificity values of 87% and 91%, respectively. In addition, Cohen’s *d* effect size values are provided in Table 3. The large Cohen’s *d* values and an estimated power of > 0.90 bolster the reliability of the sample sizes exhibited here [25, 26]. Test metrics using a lower resolution, single quadrupole desk-top ESI mass spectrometer (Advion Expression CMS) are also given in Table 2, and could discriminate groups but with somewhat lower test metrics and Cohen’s *d* values.

**Table 2:** Binary patient group SCG vs IE comparison test metrics.

**I Test Metrics (group 1 vs. group 2)**		**% LOOCV Mean (SD) group 1**	**% LOOCV** **Mean (SD)** **group 2**		**True Positive group 1**	**False Positive group 2**	**True Negative** **group 2**	**False Negative group 1**	**N**	**Figure #**
**Ion-Trap MS LOOCV Sets:**										
**SCG vs IE**		59.99% (4.71%)	39.88% (5.97%)		29/29 (100%)	1/29 (3%)	29/29 (97%)	0/29 (0%)	IE, N = 29 SCG, N = 29	2A
**Training Sets:**										
**Training: IE vs SCG**		56.71% (5.17%)	36.91% (5.83%)		20/23 (86.95%)	2/23 (8.69%)	21/23 (91.30%)	3/23 (13.04%)	IE, N = 23 SCG, N = 23	3A
**Blind Sample Sets:**										
**Blind Samples: IE vs SCG**		54.21% (1.86%)	45.35% (2.32%)		6/6 (100%)	1/6 (20%)	5/6 (80%)	0/6 (0%)	IE, N = 6 SCG, N = 6	3C
**Desktop MS LOOCV**										
**SCG vs IE**		53.40% (7.17%)	33.77% (6.00%)		27/29 (93.1%)	6/29 (3%)	28/29 (97%)	2/29 (6.9%)	SCG, N = 29 IE, N = 29	S1A
**Training Sets SCG vs IE**		66.05% (7.24%)	49.95% (8.69%)		22/25 (88%)	4/25 (16%)	21/25 (84%)	3/25 (12%)	SCG, N = 25 IE, N = 25	S1C
**Blind Samples SCG vs IE**		65.00% (6.77%)	52.50% (2.04%)		4/4 (100%)	0/4 (0%)	4/4 (100%)	0/4 (0%)	SCG, N = 45 IE, N = 4	S1D
**II Test Metrics**	**Sensitivity**	**Efficiency/** **[accuracy]**	**True Positive** **Rate**	**False Positive** **Rate**	**Specificity**	**P-value**	**Random database** **P-value**	**Cohen’s d**	**ROC (Area)**	**Figure #**
**Ion-Trap MS LOOCV Databases**										
**IE, N = 29 vs SCG, N = 29**	1	0.98	1	0.03	0.97	4.7 X 10−^20^	0.07	3.73	0.99	2A
**Training Databases:**										
**Training: IE, N = 24 vs SCG, N = 24**	1	1	1	0	1	7.0 x 10−^16^	0.06	4.45	1	3A
**Blind Samples Sets:**										
**Blind: IE, N = 6 vs SCG N = 6**	0.87	0.89	0.87	0.09	0.91	1.7 x 10−^5^	na	4.21	0.98	3C
**Desktop MS LOOCV**										
**SCG, N = 29 vs IE, N = 29**	0.93	0.95	0.93	0.03	0.97	3.6 X 10−^16^	0.05	2.96	0.99	S1A
**Training Sets SCG vs IE**	0.88	0.86	0.88	0.16	0.84	2.8 X 10−^9^	0.327	2.01	0.909	S1C
**Blind SCG vs Blind IE**	1	1	1	0	1	0.014	-	2.5	1	S1D

LOOCV (Leave One Out Cross Validation); SCG (Solitary Cysticercus Granuloma); IE (Idiopathic Epilepsy); na (not applicable); ^a^ blind SCG vs Training set SCG; ^b^ blind SCG vs Training set Control; ^c^ blind IE vs Training set IE; ^d^ blind IE vs Training set Control

**Table 3:** MS/MS range analysis of protein/peptides observed increased in SCG patient serum relative to IE.

**Panel** **I**	**Protein/Peptides Observed Increased in SCG Patient Serum Relative to IE**				
**Symbol**	**#Sera SCG (hits SCG): #Sera IE (hits IE)**	**Symbol**	**#Sera SCG (hits SCG): #Sera IE (hits IE)**	**Symbol**	**#Sera SCG (hits SCG): #Sera IE (hits IE)**
MEGF11^1^	6 (63): 2 (21)	DGKK	4 (24): 0 (0)	SI^2^	3 (46): 0 (0)
MEGF10^1^	6 (46): 3 (17)	TOP3B^1^^,^^3^	4 (23): 0 (0)	TMEM99	3 (45): 0 (0)
ADAMTS20^1^^,^^2^	5 (50): 2 (24)	FGD6^1^^,^^2^	4 (18): 0 (0)	TEX101	3 (44): 1 (5)
NOTCH2NL^1^^,^^2^	5 (43): 1 (2)	VWF^1^^,^^2^^,^^3^	3 (160): 1 (3)	BCL9L	3 (42): 1 (7)
LAMA1^1^^,^^2^	4 (102): 1 (7)	PCDHB15^1^	3 (121): 0 (0)	MACF1^1^	3 (41): 0 (0)
CACNA2D2^1^^,^^3^	4 (87): 0 (0)	ATP1A1	3 (95): 1 (15)	DMBT1^2^	3 (40): 0 (0)
STAB2^1^	4 (58): 2 (14)	AKR1B1^1^^,^^2^	3 (82): 0 (0)	MAP4K1^2^	3 (38): 0 (0)
ADGB^1^	4 (55): 2 (12)	LTBP1	3 (74): 0 (0)	USP24^1^	3 (38): 1 (5)
HIPK1^1^	4 (45): 1 (21)	CCDC129	3 (71): 1 (24)	ARMCX4	3 (37): 0 (0)
THBS1^1^^,^^3^	4 (42): 2 (7)	KIF1A^1^^,^^3^	3 (53): 1 (24)	BTBD9^1^	3 (37): 0 (0)
VLDLR^1^^,^^3^	4 (36): 2 (18)	TCTN1^1^	3 (52): 1 (9)	C8A^2^	3 (37): 0 (0)
BCORL1^1^	4 (30): 1 (11)	CCND3^2^	3 (50): 0 (0)	HELZ2^2^	3 (35): 1 (13)
THSD7B	4 (27): 1 (5)	KLK7^2^	3 (50): 0 (0)		
NOTCH2	4 (26): 1 (3)	KIAA1683	3 (48): 0 (0)		
**Panel II**	**Protein/Peptides Observed Decreased in SCG Patient Serum Relative to IE**				
**Symbol**	**#Sera SCG (hits SCG): #Sera IE (hits IE)**	**Symbol**	**#Sera SCG (hits SCG): #Sera IE (hits IE)**	**Symbol**	**#Sera SCG (hits SCG): #Sera IE (hits IE)**
IGHA2^2^	5 (22): 2 (114)	CLINT1^1^	1 (15): 4 (46)	NAV1^1^	0 (0): 3 (65)
PCM1^2^	1 (26): 5 (72)	LRP2^1^	2 (10): 4 (45)	TNC	0 (0): 3 (62)
SLIT2^1^^,^^2^^,^^3^	0 (0): 5 (65)	CEP152^1^	0 (0): 4 (43)	IARS	1 (17): 3 (56)
OTOGL^1^	1 (5): 5 (39)	ALPK2	1 (11): 4 (38)	ADGRE2^2^	0 (0): 3 (52)
LTBP2	2 (14): 5 (37)	OCA2^1^^,^^2^^,^^3^	1 (9): 4 (32)	ADAMTS18	0 (0): 3 (50)
MT-ND1^1^^,^^3^	1 (3): 5 (34)	AKAP11^1^^,^^2^^,^^3^	0 (0): 4 (29)	JAG2^1^	1 (11): 3 (48)
ARAP1^2^	0 (0): 4 (137)	PKD1^2^	2 (12): 4 (27)	DOCK10^1^^,^^2^	1 (3): 3 (46)
USP19^2^	0 (0): 4 (135)	DACT2^1^	2 (10): 4 (22)	FRY	1 (12): 3 (45)
CTCFL	0 (0): 4 (126)	FBXL17^1^	1 (3): 4 (13)	LAMB3	0 (0): 3 (43)
LAMA3^2^	2 (30): 4 (102)	LCE1A	0 (0): 3 (178)	MT-ND2^1^^,^^2^	1 (3): 3 (43)
SCARF1^2^	1 (25): 4 (86)	ADAM11^1^^,^^2^^,^^3^	0 (0): 3 (87)	LINC02280	0 (0): 3 (40)
LAMA2^1^^,^^2^	0 (0): 4 (67)	MAOB^1^^,^^3^	0 (0): 3 (68)	KALRN^1^	1 (13): 3 (39)
EPHB2^1^^,^^2^^,^^3^	1 (13): 4 (62)	BIRC6	1 (15): 3 (66)		
CNOT10	0 (0): 4 (52)	VPS13D^1^^,^^3^	0 (0): 3 (66)		

^1^neurological

^2^immune/inflammation

^3^seizure related (shaded). This table represents proteins identified by MS/MS having a serum number and MS/MS “hit” ratio of at least a 2 fold change or greater.

### Phenotype assessment of SCG and IE patients using tandem MS/MS of serum peptide/proteins and bioinformatics cell pathway/disease/function analysis

The mass peak tandem MS/MS analysis of 10 SCG and 10 IE sera in a range between 900 and 1008 m/z identified a total of 262 peptides/proteins in three or more patient sera out of 10 in either group (exhibited in S2 Table). In order to focus on peptides/proteins with the largest differences between the two groups, we identified a subset of 164 peptides/proteins showing at least a two-fold difference in number of hits and number of positive sera between SCG and IE groups and which were present in three or more sera per group with two or more unique peptides identifying each peptide/protein. From this subset of 164 peptides/proteins, 80 peptides/proteins with the greatest numbers of MS/MS peptide sequence “hits” and sera number presence are shown in Table 3. This Table exhibits peptides/proteins expressed differently between the SCG and IE sera groups, those increased in SCG sera relative to IE sera (Panel I) and those increased in IE sera relative to SCG sera (Panel II). It is important to report observing peptides/proteins either as elevated or depressed relative to the SCG disease state at this early stage of study as either change could be resulting from a SCG disease-significant events.

A PubMed/Medline literature search showed 53% of these differentially expressed proteins were related to neurological function, 36% to immune/inflammation, and 19% to seizures. Notable epilepsy/seizure related proteins (shaded cells in Table 3) include CACNA2D2 [30], THBS1 [31], VLDLR [30, 32], THBS1 [31], VLDLR [32], TOP3B [33], VWF [34, 35], KIF1A [36], SLIT2 [37], MT-ND1 [38], EPHB2 [39], OCA2 [40], AKAP11 [41], ADAM11 [42], MAOB [43], and VPS13D [44].

**Fig 4:**
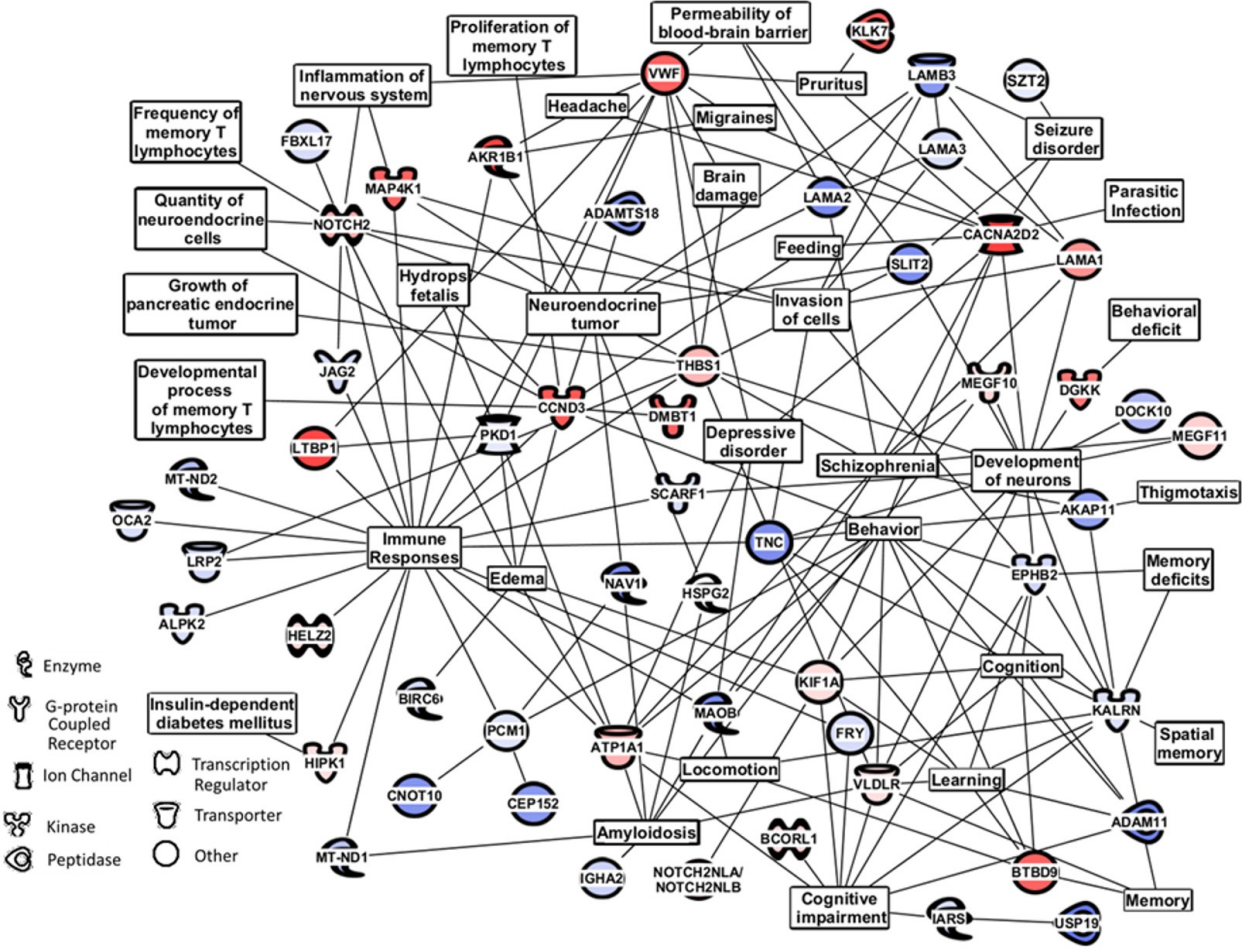
IPA m/z Range MS/MS Serum Data Analysis for SCG vs IE Patients. The 80 proteins shown in Table 3 having a 2x ratio difference for both the number of hits and the number of sera observed were analyzed using bioinformatics software. Affected physiological/cellular pathways and serum protein assignments from Table 3 were found to distinguish SCG from IE individuals. The top 80 proteins having a 2x ratio difference for both the number of hits and the number of sera observed were used for this analysis. Analysis performed with Ingenuity Pathway Analysis (IPA) bioinformatics software (Qiagen, Inc.).

The 80 proteins/peptides expressed differently between the SCG and IE groups were also analyzed by Ingenuity Pathway Analysis (IPA) to identify affected networks of cellular/biochemical pathways/systems (Fig 4). For this analysis the important seizure protein SZT2 from S1 Table was also included. Use of the MS/MS peptide “hits’ in IPA is further described in S11 File, Remarks Concerning Methods. Pathways affected include immune responses, neurology, amyloidosis and cognition, behavior, and seizure. Blood brain barrier, brain damage, edema, and headache pathway effects, as well as a potential schizophrenia nodes with connections to MEGF10 and MEGF11 which binds Atrophin 1 (ATN1) a seizure related protein also appear [6, 45–49]. Of note, some of the proteins listed in Table 3 and Fig 4 may not have a direct connection to disease states observed, but serve as connections to other biomolecular pathways of interest like behavior and other neuroresponses, potentially resulting from brain damage. S2 Fig shows a focused IPA pathway analysis of the VWF and CACNA2D2 proteins. Of interest, various inflammatory component(s) and cell death (necrosis) including neuronal cell death become evident here. S3 Fig demonstrates changes observed related to small molecule and ion transport pathways which are an important part of both normal cellular and nerve cell function. These IPA analysis gives a potential overview of the cell pathways at the protein level undergoing change upon comparison of sera from SCG patients and IE patients with their respective disease states.

## Discussion

Examination of biomolecules in peripheral blood, e.g. peptides/proteins, that change with disease states like SCG or IE could provide diagnostic, phenotypic, mechanistic, and therapeutic insights into these disease states. The results presented here are the first report using serum mass peak profiling to help discriminate and help identify biochemically these two specific patient populations that are, otherwise, indistinguishable on clinical evaluation. Mass peak profiling of sera using ESI-MS and LOOCV analysis identified mass peaks significantly changing upon comparison of patients with SCG or IE. Randomization of serum samples resulted in loss of discrimination between the groups, suggesting there exists a physiological basis for the observed patient group discriminatory results. Notably, the sensitivity test metric (true positive rate) of this LOOCV analysis exceeded that of serum EITB for SCG [13, 50]. It is likely the positive results of these studies are due to the large number of different identifiers (mass peaks) applied by this approach, as opposed to more specific tests such as EITB and antigen ELISA [19, 20, 51]. Results reported here using ESI-MS were substantiated using an instrument with a different mass analyzer of lower resolution (Fig 3). These results support the hypothesis that the disease states induce biomolecular alterations that are reflected in the peripheral blood and have a role in identifying specific clinical groups [13, 19, 20, 52]. This work focuses on the SCG and IE disease states and adds to our previous study using the ESI-MS platform that was able to distinguish the serum mass profile of a heterogeneous group of NCC (solitary, multiple, calcified, and healed cyst[s]) patients from individuals with IE [13].

The LOOCV mass peaks being analysed in this study, e.g. those approximately 500 to 1200 m/z, likely include the lower mass peptide “serome”, biomolecules resulting from host tissue/organ exoprotease activities and other cell/tissue signaling activities [53, 54]. To aid in identifying specific physiological differences in this complex biomolecular milieu, MS/MS structure determinations were performed. The identification of differentially expressed biomolecules and biochemical pathways could be helpful in developing novel diagnostic biomarkers and/or therapeutics. For this, this study conducted a range analysis (900–1008 m/z) revealing a prominent epilepsy phenotype with 15 of the 80 different proteins (19%) with known associations to epilepsy (Table 3). For example, seizure related proteins CACNA2D2 (calcium voltage-gated channel auxiliary subunit alpha 2 delta 2) and VWF (von Willebrand factor) were found primarily in SCG patient sera. CACNA2D2 is an important epilepsy-related calcium channel protein involved in small molecule ligand interactions as well as neuronal cell death pathways [30] VWF is involved in inflammation, seizures, neurodegeneration, and blood brain barrier permeability [34, 35]. Thrombospondin-1 (THBS1) and DNA topoisomerase 3-beta-1 (TOP3B), proteins reported to be associated with various forms of epilepsy [31, 33, 55], were also over-represented in SCG patients compared with IE [31, 33, 55]. Other notable findings included higher observation in the IE group compard to the SCG group of SLIT2 (Slit Guidance Ligand 2 protein), an extracellular matrix protein and member of the SLIT family of proteins which has a role(s) in the blood brain barrier (BBB) integrity [37, 56] The mitochondrial protein MT-ND1 with epilepsy background was also found to be observed more in the IE group compared to the SCG group (40).

Less stringent focus on differentially expressed proteins/peptides (removal of the 2x sera and MS/MS “hit” differential window in Table 3 and Fig 4) widened the scope of potentially relevant findings. Such examples include SZT2 (Seizure Threshold 2 Protein) [57], which was identified primarily in IE sera (S2 Table). SZT2 is highly expressed in the brain and appears to enhance epileptogenesis [57]. Other mitochondrial proteins with epilepsy backgrounds that were observed more often in the IE group included MT-CYB and MT-ND5. In contrast, no *T*. *solium* peptides/proteins were identified using a criteria of at least three sera out of 10 suggesting this study mainly detected host responses against *T*. *solium* infection or IE pathology. All these relate to an overall epilepsy phenotype with additional observations from this study. In this regard, IPA analysis of differentially expressed proteins/peptides highlighted the importance of inflammation, neurological damage, blood brain barrier effects, cognition, cell death, behavior, and seizure-related pathway changes taking place in patients with SCG compared to patients with IE. Inflammatory and neurological responses have been previously documented in both NCC and SCG-related epilepsy as well as IE [1, 12, 50]. Finding proteins/peptides that are known to be related to seizure phenotypes in patients with SCG and IE lends credence to the ability of the mass profiling platform and methodology described here to help decipher these pathologies. Future studies will examine larger numbers of serum samples in these contexts, and also test for peptide/protein presences in sera using immunoassays. Such analyses are difficult in the context of the present study since mostly peptides are being identified here by MS/MS, and matching available antibodies and their epitopes to such peptides is a “hit and miss” process that likely will involve de novo acquisitions of peptide-specific antibodies.

## Supporting information

S1 Fig IPA m/z range MS/MS serum data analysis from Table 3 for SCG vs IE patients involving neuronal cell death and the epilepsy-associated calcium channel protein CACNA2D2. (TIF)

S2 Fig PA m/z range MS/MS serum data analysis from Table 3 for SCG vs IE patients involving transport of small molecules and Ions. (TIF)

S3 Fig A Focused IPA m/Z Range MS/MS serum data analysis, from Table 3, for SCG vs IE patients involving transport of small molecules Ion transport potentially related to nervous function. (DOCX)

S1 Table MS/MS results of range analysis 3 or better sera in either group. (DOCX)

S2 Table Patients used for each figure. (DOCX)

S3 Table Peaks utilized for each figure. (DOCX)

S1 File LCQ ADVANTAGE extracted MS spectral data. (XLSX)

S2 File LCQ ADVANTAGE normalized MS spectral data. (XLSX)

S3 File LCQ ADVANTAGE peaked MS spectral data. (XLSX)

S4 File LCQ ADVANTAGE normalized MS peak data. (XLSX)

S5 File LCQ ADVANTAGE mean MS peak data normalized. (XLSX)

S6 File Advion extracted MS spectral data. (XLSX)

S7 File Advion compressed data to 1 Dalton m/z. (XLSX)

S8 File Advion normalized MS spectral data. (XLSX)

S9 File Advion peaked MS spectral data. (XLSX)

S10 File Advion normalized MS peak data. (XLSX)

S11 File Advion mean MS peak data normalized. (XLSX)

S12 File Remarks concerning methods. (DOCX)

S1 Data Advion MS acquisition files: Patients 1–3. (ZIP)

S2 Data Advion MS acquisition files: Patients 4–5. (ZIP)

S3 Data Advion MS acquisition files: Patients 7–9. (ZIP)

S4 Data Advion MS acquisition files: Patients 10–12. (ZIP)

S5 Data Advion MS acquisition files: Patients 13–15. (ZIP)

S6 Data Advion MS acquisition files: Patients 16–18. (ZIP)

S7 Data Advion MS acquisition files: P[atients 19–21. (ZIP)

S8 Data Advion MS acquisition files: Patients 22–23. (ZIP)

S9 Data Advion MS acquisition files: Patients 25–27. (ZIP)

S10 Data Advion MS acquisition files: Patients 28–30. (ZIP)

S11 Data Advion MS acquisition files: Patients 31–32. (ZIP)

S12 Data Advion MS acquisition files: Patients 34–35. (ZIP)

S13 Data Advion MS acquisition files: Patients 37–39. (ZIP)

S14 Data Advion MS acquisition file: Patients 40–42. (ZIP)

S15 Data Advion MS acquisition files: Patients 43–45. (ZIP)

S16 Data Advion MS acquisition files: Patients 46–48. (ZIP)

S17 Data Advion MS acquisition files: Patients 49–51. (ZIP)

S18 Data Advion MS acquisition files: Patients 52–54. (ZIP)

S19 Data Advion MS acquisition files: Patients 55–57. (ZIP)

S20 Data Advion MS acquisition files: Patients 58–60. (ZIP)

S21 Data Advion MS acquisition files: Patients 6 & 24. (ZIP)

S22 Data Advion MS acquisition files: Patient 33. (ZIP)

S23 Data Advion MS acquisition files: Patient 36. (ZIP)

S24 Data LCQ MS acquisition files: Patients 1–4. (ZIP)

S25 Data LCQ MS acquisition files: Patients 5–7. (ZIP)

S26 Data LCQ MS acquisition files: Patients 8–11. (ZIP)

S27 Data LCQ MS acquisition files: Patients 12–15. (ZIP)

S28 Data LCQ MS acquisition files: Patients 16–20. (ZIP)

S29 Data LCQ MS acquisition files: Patients 21–24. (ZIP)

S30 Data LCQ MS acquisition files: Patients 25–28. (ZIP)

S31 Data CQ MS acquisition files: Patients 29–33. (ZIP)

S32 Data LCQ MS acquisition files: Patients 34–38. (ZIP)

S33 Data LCQ MS acquisition files: Patients 39–43. (ZIP)

S34 Data LCQ MS acquisition files: Patients 44–48. (ZIP)

S35 Data LCQ MS acquisition files: Patients 49–53. (ZIP)

S36 Data LCQ MS acquisition files: patients 54–58. (ZIP)

S37 Data LCQ MSMS range acquisition files: patients:1, 2, 6, 7, 10, 11, 15 & 23. (ZIP)

S38 Data LCQ MSMS range acquisition files: patients: 24, 30, 31, 32, 33 & 34. (ZIP)

S39 Data LCQ MSMS range acquisition files: patients: 3, 37, 39, 47, 48 & 58. (ZIP)
