# Supplementary figures and images for: Distinguishing patients with idiopathic epilepsy from solitary cysticercus granuloma epilepsy and biochemical phenotype assessment using a serum biomolecule profiling platform
Source: PLoS One. 2020 Aug 21;15(8):e0237064. doi: 10.1371/journal.pone.0237064 (PMC7527271; doi:10.1371/journal.pone.0237064)

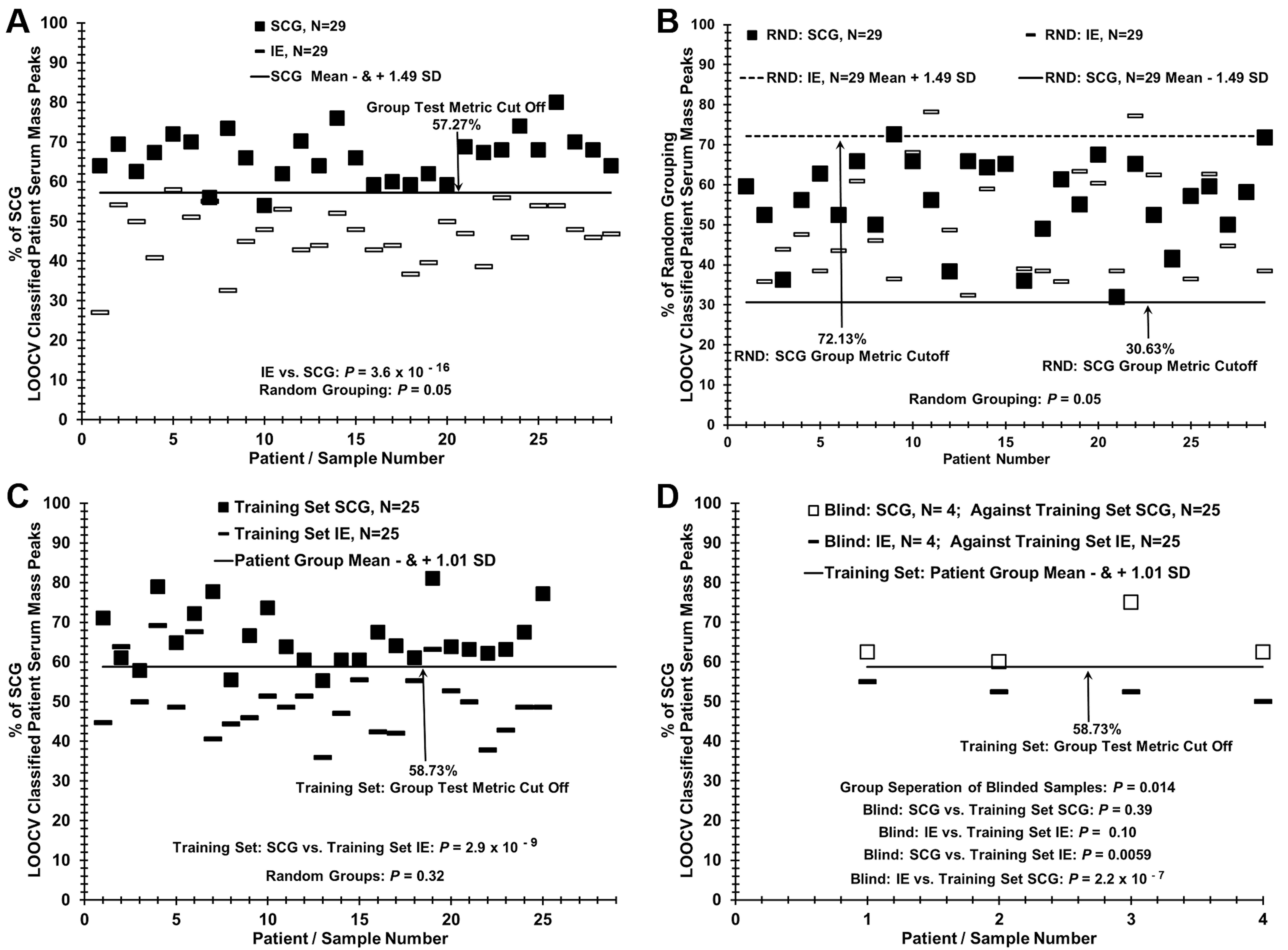

Supplement: S1 Fig — (TIF) [file pone.0237064.s001.tif]

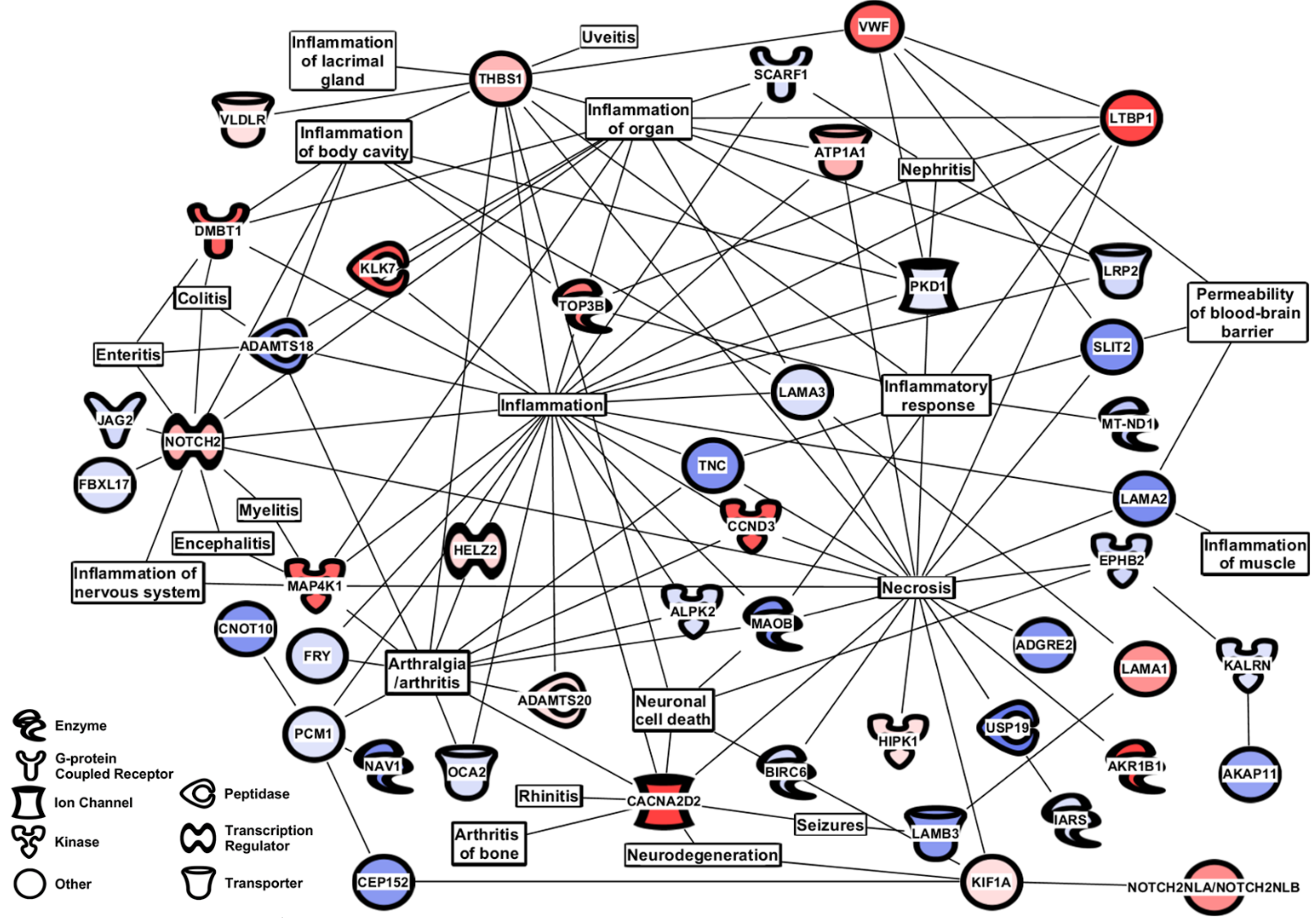

Supplement: S2 Fig — (TIF) [file pone.0237064.s002.tif]
