# Supplementary material for: Distinguishing patients with idiopathic epilepsy from solitary cysticercus granuloma epilepsy and biochemical phenotype assessment using a serum biomolecule profiling platform
Source: PLoS One. 2020 Aug 21;15(8):e0237064. doi: 10.1371/journal.pone.0237064 (PMC7527271; doi:10.1371/journal.pone.0237064)
Supplement: S3 Fig — (DOCX) [file pone.0237064.s003.docx]

**S3 Fig. A Focused IPA m/Z Range MS/MS Serum Data Analysis, From Table 3, for SCG vs IE Patients Involving transport of small molecules Ion transport potentially related to nervous function.**

**
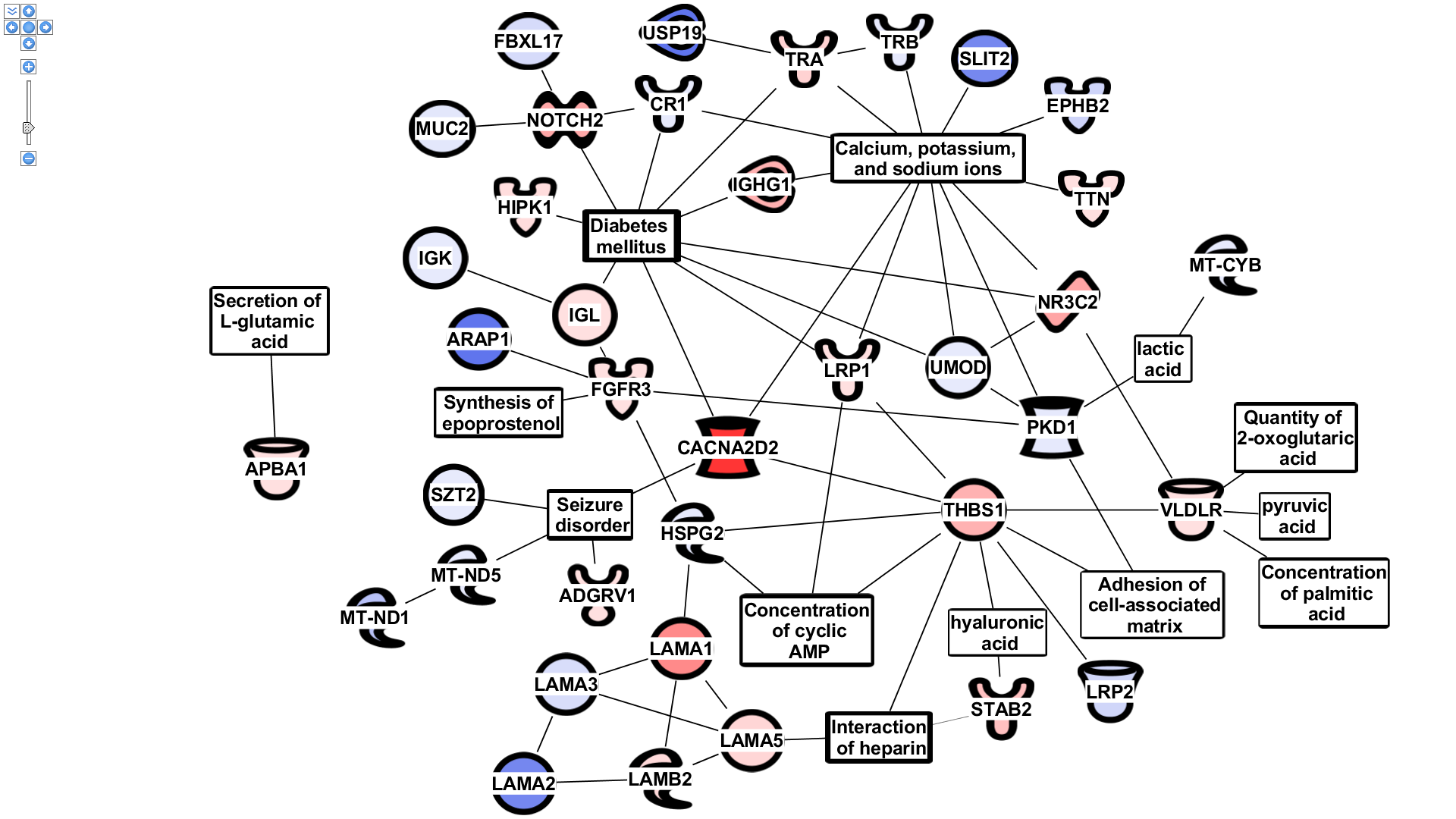
**

**S3 Fig. Legend:** Molecular ion transport pathways are presented with variations a result of differences between components identified from sera of SCG and IE patients. Of interest, physiologic pathways associated with nervous system, cellular energetics and signal transduction are represented. Proteins in this pathway have been identified by range MS/MS analysis having 3 or more sera and a hit and sera ratio greater than 2X.
