# Supplementary material for: Distinguishing patients with idiopathic epilepsy from solitary cysticercus granuloma epilepsy and biochemical phenotype assessment using a serum biomolecule profiling platform
Source: PLoS One. 2020 Aug 21;15(8):e0237064. doi: 10.1371/journal.pone.0237064 (PMC7527271; doi:10.1371/journal.pone.0237064)
Supplement: S1 Table — (DOCX) [file pone.0237064.s004.docx]

**S2 Table. MS/MS results of range analysis 3 or better sera in either group.**

| order | Peptide/Protein Identified | #sera SCG Total ( SCG Total ) : #sera IE Total ( IE Total ) : (if both sera and hits show 2x ratio then running total of 2x protein # provided or (T=true; F=False) 2x in SCG : 2x IE | Ratio Submitted to IPA =imlog2( (#hits SCG +1) / (#hits IE+1) ) |
| --- | --- | --- | --- |
| 1 | IGH | 10 ( 482 ) : 10 ( 923 ) : F : F | -0.9359 |
| 2 | IGL | 9 ( 314 ) : 10 ( 255 ) : F : F | 0.2992 |
| 3 | TTN | 9 ( 224 ) : 9 ( 221 ) : F : F | 0.0194 |
| 4 | IGK | 9 ( 102 ) : 8 ( 108 ) : F : F | -0.0817 |
| 5 | TRB | 8 ( 149 ) : 7 ( 321 ) : F : T | -1.1021 |
| 6 | EBF4 | 8 ( 301 ) : 8 ( 295 ) : F : F | 0.029 |
| 7 | MUC5AC | 5 ( 66 ) : 8 ( 184 ) : F : T | -1.4653 |
| 8 | CR1 | 5 ( 87 ) : 7 ( 99 ) : F : F | -0.1844 |
| 9 | APBA1 | 7 ( 80 ) : 7 ( 77 ) : F : F | 0.0544 |
| 10 | NR3C2 | 6 ( 120 ) : 5 ( 16 ) : F : T | 2.8314 |
| 11 | MUC2 | 6 ( 38 ) : 4 ( 89 ) : F : T | -1.2065 |
| 12 | MUC17 | 4 ( 23 ) : 6 ( 83 ) : F : T | -1.8074 |
| 13 | MEGF11 | 6 ( 63 ) : 2 ( 21 ) : 1 | 1.5406 |
| 14 | FBN3 | 3 ( 38 ) : 6 ( 62 ) : F : F | -0.6919 |
| 15 | MEGF10 | 6 ( 46 ) : 3 ( 17 ) : F : T | 1.3847 |
| 16 | MUC16 | 4 ( 27 ) : 6 ( 28 ) : F : F | -0.0506 |
| 17 | IGHA2 | 5 ( 22 ) : 2 ( 114 ) : 2 | -2.3219 |
| 18 | FBN2 | 5 ( 95 ) : 4 ( 36 ) : F : T | 1.3755 |
| 19 | FAT4 | 5 ( 44 ) : 4 ( 83 ) : F : F | -0.9005 |
| 20 | PCM1 | 1 ( 26 ) : 5 ( 72 ) : 3 | -1.4349 |
| 21 | ADGRV1 | 5 ( 67 ) : 3 ( 42 ) : F : F | 0.6612 |
| 22 | SLIT2 | 0 ( 0 ) : 5 ( 65 ) : 4 | -6.0444 |
| 23 | ADAMTS20 | 5 ( 50 ) : 2 ( 24 ) : 5 | 1.0286 |
| 24 | LOC100996763/NOTCH2NL | 5 ( 43 ) : 1 ( 2 ) : 6 | 3.8745 |
| 25 | TRA | 5 ( 39 ) : 3 ( 14 ) : F : T | 1.415 |
| 26 | MPRIP | 5 ( 28 ) : 2 ( 39 ) : T : F | -0.4639 |
| 27 | OTOGL | 1 ( 5 ) : 5 ( 39 ) : 7 | -2.737 |
| 28 | LTBP2 | 2 ( 14 ) : 5 ( 37 ) : 8 | -1.341 |
| 29 | MT-ND1 | 1 ( 3 ) : 5 ( 34 ) : 9 | -3.1293 |
| 30 | MT-CYB | 4 ( 27 ) : 5 ( 32 ) : F : F | -0.237 |
| 31 | FGFR3 | 5 ( 30 ) : 2 ( 25 ) : T : F | 0.2538 |
| 32 | IGHG1 | 3 ( 215 ) : 4 ( 38 ) : F : T | 2.4695 |
| 33 | ARAP1 | 0 ( 0 ) : 4 ( 137 ) : 10 | -7.1085 |
| 34 | USP19 | 0 ( 0 ) : 4 ( 135 ) : 11 | -7.0875 |
| 35 | CTCFL | 0 ( 0 ) : 4 ( 126 ) : 12 | -6.9887 |
| 36 | LAMA3 | 2 ( 30 ) : 4 ( 102 ) : F : T | -1.7323 |
| 37 | LAMA1 | 4 ( 102 ) : 1 ( 7 ) : 13 | 3.6865 |
| 38 | CACNA2D2 | 4 ( 87 ) : 0 ( 0 ) : 14 | 6.4594 |
| 39 | SCARF1 | 1 ( 25 ) : 4 ( 86 ) : 15 | -1.7425 |
| 40 | HSPG2 | 4 ( 48 ) : 4 ( 83 ) : F : F | -0.7776 |
| 41 | MUC4 | 4 ( 77 ) : 4 ( 38 ) : F : T | 1 |
| 42 | ZFP1 | 4 ( 77 ) : 3 ( 21 ) : F : T | 1.826 |
| 43 | LRP1 | 4 ( 69 ) : 3 ( 52 ) : F : F | 0.4014 |
| 44 | LAMA2 | 0 ( 0 ) : 4 ( 67 ) : 16 | -6.0875 |
| 45 | EPHB2 | 1 ( 13 ) : 4 ( 62 ) : 17 | -2.1699 |
| 46 | STAB2 | 4 ( 58 ) : 2 ( 14 ) : F : T | 1.9758 |
| 47 | SSH1 | 4 ( 55 ) : 3 ( 30 ) : F : F | 0.8532 |
| 48 | ADGB | 4 ( 55 ) : 2 ( 12 ) : F : T | 2.1069 |
| 49 | LAMA5 | 4 ( 53 ) : 3 ( 21 ) : F : T | 1.2955 |
| 50 | CNOT10 | 0 ( 0 ) : 4 ( 52 ) : 18 | -5.7279 |
| 51 | PCSK5 | 4 ( 50 ) : 3 ( 18 ) : F : T | 1.4245 |
| 52 | ZNF469 | 4 ( 20 ) : 3 ( 47 ) : F : T | -1.1926 |
| 53 | CLINT1 | 1 ( 15 ) : 4 ( 46 ) : 19 | -1.5546 |
| 54 | LRP2 | 2 ( 10 ) : 4 ( 45 ) : F : T | -2.0641 |
| 55 | HIPK1 | 4 ( 45 ) : 1 ( 21 ) : 20 | 1.0641 |
| 56 | LAMB2 | 4 ( 44 ) : 3 ( 19 ) : F : T | 1.1699 |
| 57 | PEAR1 | 3 ( 14 ) : 4 ( 44 ) : F : T | -1.585 |
| 58 | SYNE2 | 4 ( 43 ) : 2 ( 27 ) : F : F | 0.6521 |
| 59 | CEP152 | 0 ( 0 ) : 4 ( 43 ) : 21 | -5.4594 |
| 60 | THBS1 | 4 ( 42 ) : 2 ( 7 ) : F : T | 2.4263 |
| 61 | SVEP1 | 3 ( 35 ) : 4 ( 40 ) : F : F | -0.1876 |
| 62 | ALPK2 | 1 ( 11 ) : 4 ( 38 ) : 22 | -1.7004 |
| 63 | VLDLR | 4 ( 36 ) : 2 ( 18 ) : F : F | 0.9615 |
| 64 | OTOG | 3 ( 20 ) : 4 ( 35 ) : F : F | -0.7776 |
| 65 | SZT2 | 2 ( 24 ) : 4 ( 34 ) : F : F | -0.4854 |
| 66 | CEP350 | 3 ( 26 ) : 4 ( 33 ) : F : F | -0.3326 |
| 67 | OCA2 | 1 ( 9 ) : 4 ( 32 ) : 23 | -1.7225 |
| 68 | NACA | 4 ( 31 ) : 4 ( 14 ) : F : T | 1.0931 |
| 69 | DNAH14 | 3 ( 30 ) : 4 ( 30 ) : F : F | 0 |
| 70 | UBR4 | 4 ( 30 ) : 1 ( 29 ) : T : F | 0.0473 |
| 71 | BCORL1 | 4 ( 30 ) : 1 ( 11 ) : 24 | 1.3692 |
| 72 | AKAP11 | 0 ( 0 ) : 4 ( 29 ) : 25 | -4.9069 |
| 73 | PKD1 | 2 ( 12 ) : 4 ( 27 ) : F : T | -1.1069 |
| 74 | THSD7B | 4 ( 27 ) : 1 ( 5 ) : 26 | 2.2224 |
| 75 | NOTCH2 | 4 ( 26 ) : 1 ( 3 ) : 27 | 2.7549 |
| 76 | DGKK | 4 ( 24 ) : 0 ( 0 ) : 28 | 4.6439 |
| 77 | ADGRB1 | 2 ( 23 ) : 4 ( 12 ) : F : F | 0.8845 |
| 78 | TOP3B | 4 ( 23 ) : 0 ( 0 ) : 29 | 4.585 |
| 79 | DACT2 | 2 ( 10 ) : 4 ( 22 ) : F : T | -1.0641 |
| 80 | LOC107985428 | 4 ( 21 ) : 2 ( 20 ) : F : F | 0.0671 |
| 81 | MT-ND5 | 3 ( 11 ) : 4 ( 20 ) : F : F | -0.8074 |
| 82 | FGD6 | 4 ( 18 ) : 0 ( 0 ) : 30 | 4.2479 |
| 83 | UMOD | 1 ( 11 ) : 4 ( 13 ) : T : F | -0.2224 |
| 84 | FBXL17 | 1 ( 3 ) : 4 ( 13 ) : 31 | -1.8074 |
| 85 | AR | 2 ( 53 ) : 3 ( 203 ) : F : T | -1.9175 |
| 86 | LCE1A | 0 ( 0 ) : 3 ( 178 ) : 32 | -7.4838 |
| 87 | VWF | 3 ( 160 ) : 1 ( 3 ) : 33 | 5.3309 |
| 88 | PCDHB15 | 3 ( 121 ) : 0 ( 0 ) : 34 | 6.9307 |
| 89 | WNK1 | 2 ( 14 ) : 3 ( 114 ) : F : T | -2.9386 |
| 90 | ATP1A1 | 3 ( 95 ) : 1 ( 15 ) : 35 | 2.585 |
| 91 | ITGB6 | 3 ( 92 ) : 3 ( 70 ) : F : F | 0.3894 |
| 92 | HECTD4 | 3 ( 22 ) : 2 ( 91 ) : F : T | -2 |
| 93 | SEC16A | 2 ( 90 ) : 3 ( 35 ) : F : T | 1.3379 |
| 94 | ADAM11 | 0 ( 0 ) : 3 ( 87 ) : 36 | -6.4594 |
| 95 | AKR1B1 | 3 ( 82 ) : 0 ( 0 ) : 37 | 6.375 |
| 96 | MAP1B | 3 ( 74 ) : 3 ( 40 ) : F : F | 0.8713 |
| 97 | LTBP1 | 3 ( 74 ) : 0 ( 0 ) : 38 | 6.2288 |
| 98 | CCDC129 | 3 ( 71 ) : 1 ( 24 ) : 39 | 1.5261 |
| 99 | ZNF106 | 2 ( 70 ) : 3 ( 53 ) : F : F | 0.3949 |
| 100 | MAOB | 0 ( 0 ) : 3 ( 68 ) : 40 | -6.1085 |
| 101 | BIRC6 | 1 ( 15 ) : 3 ( 66 ) : 41 | -2.0661 |
| 102 | VPS13D | 0 ( 0 ) : 3 ( 66 ) : 42 | -6.0661 |
| 103 | SIDT2 | 3 ( 65 ) : 1 ( 50 ) : T : F | 0.372 |
| 104 | NAV1 | 0 ( 0 ) : 3 ( 65 ) : 43 | -6.0444 |
| 105 | MUC3A | 3 ( 22 ) : 3 ( 64 ) : F : T | -1.4988 |
| 106 | NFX1 | 1 ( 62 ) : 3 ( 54 ) : T : F | 0.1959 |
| 107 | TNC | 0 ( 0 ) : 3 ( 62 ) : 44 | -5.9773 |
| 108 | NOTCH4 | 3 ( 43 ) : 3 ( 60 ) : F : F | -0.4713 |
| 109 | IARS | 1 ( 17 ) : 3 ( 56 ) : 45 | -1.663 |
| 110 | DSCAML1 | 3 ( 55 ) : 2 ( 8 ) : F : T | 2.6374 |
| 111 | KIF1A | 3 ( 53 ) : 1 ( 24 ) : 46 | 1.111 |
| 112 | TCTN1 | 3 ( 52 ) : 1 ( 9 ) : 47 | 2.406 |
| 113 | ADGRE2 | 0 ( 0 ) : 3 ( 52 ) : 48 | -5.7279 |
| 114 | ADAMTS18 | 0 ( 0 ) : 3 ( 50 ) : 49 | -5.6724 |
| 115 | CCND3 | 3 ( 50 ) : 0 ( 0 ) : 50 | 5.6724 |
| 116 | KLK7 | 3 ( 50 ) : 0 ( 0 ) : 51 | 5.6724 |
| 117 | JAG2 | 1 ( 11 ) : 3 ( 48 ) : 52 | -2.0297 |
| 118 | KIAA1683 | 3 ( 48 ) : 0 ( 0 ) : 53 | 5.6147 |
| 119 | DHX34 | 3 ( 46 ) : 2 ( 6 ) : F : T | 2.7472 |
| 120 | DOCK10 | 1 ( 3 ) : 3 ( 46 ) : 54 | -3.5546 |
| 121 | SI | 3 ( 46 ) : 0 ( 0 ) : 55 | 5.5546 |
| 122 | DPYD | 3 ( 45 ) : 2 ( 7 ) : F : T | 2.5236 |
| 123 | FRY | 1 ( 12 ) : 3 ( 45 ) : 56 | -1.8231 |
| 124 | TMEM99 | 3 ( 45 ) : 0 ( 0 ) : 57 | 5.5236 |
| 125 | JAG1 | 3 ( 44 ) : 2 ( 21 ) : F : T | 1.0324 |
| 126 | TEX101 | 3 ( 44 ) : 1 ( 5 ) : 58 | 2.9069 |
| 127 | SLIT1 | 2 ( 43 ) : 3 ( 20 ) : F : T | 1.0671 |
| 128 | TNXA | 3 ( 43 ) : 1 ( 23 ) : T : F | 0.8745 |
| 129 | MT-ND2 | 1 ( 3 ) : 3 ( 43 ) : 59 | -3.4594 |
| 130 | LAMB3 | 0 ( 0 ) : 3 ( 43 ) : 60 | -5.4594 |
| 131 | BCL9L | 3 ( 42 ) : 1 ( 7 ) : 61 | 2.4263 |
| 132 | MACF1 | 3 ( 41 ) : 0 ( 0 ) : 62 | 5.3923 |
| 133 | DMBT1 | 3 ( 40 ) : 0 ( 0 ) : 63 | 5.3576 |
| 134 | LINC02280 | 0 ( 0 ) : 3 ( 40 ) : 64 | -5.3576 |
| 135 | TRPS1 | 2 ( 28 ) : 3 ( 39 ) : F : F | -0.4639 |
| 136 | KALRN | 1 ( 13 ) : 3 ( 39 ) : 65 | -1.5146 |
| 137 | ADGRF5 | 2 ( 38 ) : 3 ( 35 ) : F : F | 0.1155 |
| 138 | USP24 | 3 ( 38 ) : 1 ( 5 ) : 66 | 2.7004 |
| 139 | MAP4K1 | 3 ( 38 ) : 0 ( 0 ) : 67 | 5.2854 |
| 140 | ARMCX4 | 3 ( 37 ) : 0 ( 0 ) : 68 | 5.2479 |
| 141 | BTBD9 | 3 ( 37 ) : 0 ( 0 ) : 69 | 5.2479 |
| 142 | C8A | 3 ( 37 ) : 0 ( 0 ) : 70 | 5.2479 |
| 143 | ILF3 | 3 ( 28 ) : 3 ( 36 ) : F : F | -0.3515 |
| 144 | MUC6 | 2 ( 8 ) : 3 ( 36 ) : F : T | -2.0395 |
| 145 | HELZ2 | 3 ( 35 ) : 1 ( 13 ) : 71 | 1.3626 |
| 146 | NR1D1 | 1 ( 2 ) : 3 ( 35 ) : 72 | -3.585 |
| 147 | IGHA1 | 3 ( 33 ) : 2 ( 34 ) : F : F | -0.0418 |
| 148 | ISM2 | 1 ( 15 ) : 3 ( 34 ) : 73 | -1.1293 |
| 149 | P2RX7 | 0 ( 0 ) : 3 ( 34 ) : 74 | -5.1293 |
| 150 | PAPLN | 1 ( 22 ) : 3 ( 33 ) : T : F | -0.5639 |
| 151 | PCNX2 | 1 ( 15 ) : 3 ( 33 ) : 75 | -1.0875 |
| 152 | TP53BP2 | 1 ( 11 ) : 3 ( 33 ) : 76 | -1.5025 |
| 153 | OBSCN | 3 ( 33 ) : 1 ( 9 ) : 77 | 1.7655 |
| 154 | LOC100288966/POTED | 1 ( 5 ) : 3 ( 33 ) : 78 | -2.5025 |
| 155 | GDA | 1 ( 3 ) : 3 ( 33 ) : 79 | -3.0875 |
| 156 | ANK3 | 2 ( 23 ) : 3 ( 32 ) : F : F | -0.4594 |
| 157 | ABI3 | 3 ( 16 ) : 1 ( 32 ) : T : F | -0.9569 |
| 158 | DIDO1 | 3 ( 32 ) : 0 ( 0 ) : 80 | 5.0444 |
| 159 | HIVEP3 | 3 ( 32 ) : 0 ( 0 ) : 81 | 5.0444 |
| 160 | ITGAE | 0 ( 0 ) : 3 ( 32 ) : 82 | -5.0444 |
| 161 | ADAMTS7 | 3 ( 31 ) : 2 ( 28 ) : F : F | 0.142 |
| 162 | HNRNPM | 1 ( 17 ) : 3 ( 31 ) : T : F | -0.8301 |
| 163 | MYLK | 1 ( 14 ) : 3 ( 31 ) : 83 | -1.0931 |
| 164 | TEK | 0 ( 0 ) : 3 ( 31 ) : 84 | -5 |
| 165 | AGRN | 2 ( 11 ) : 3 ( 30 ) : F : T | -1.3692 |
| 166 | NSD3 | 2 ( 11 ) : 3 ( 30 ) : F : T | -1.3692 |
| 167 | ATP8B2 | 1 ( 11 ) : 3 ( 30 ) : 85 | -1.3692 |
| 168 | USH2A | 3 ( 30 ) : 1 ( 4 ) : 86 | 2.6323 |
| 169 | ZNF562 | 0 ( 0 ) : 3 ( 30 ) : 87 | -4.9542 |
| 170 | FCGBP | 3 ( 29 ) : 3 ( 24 ) : F : F | 0.263 |
| 171 | CREBBP | 2 ( 18 ) : 3 ( 29 ) : F : F | -0.659 |
| 172 | HDAC5 | 1 ( 29 ) : 3 ( 17 ) : T : F | 0.737 |
| 173 | ZKSCAN2 | 3 ( 29 ) : 1 ( 9 ) : 88 | 1.585 |
| 174 | C5 | 3 ( 29 ) : 0 ( 0 ) : 89 | 4.9069 |
| 175 | DNAH10 | 3 ( 29 ) : 0 ( 0 ) : 90 | 4.9069 |
| 176 | SLC4A3 | 3 ( 29 ) : 0 ( 0 ) : 91 | 4.9069 |
| 177 | ADAMTSL1 | 1 ( 5 ) : 3 ( 28 ) : 92 | -2.273 |
| 178 | COL1A2 | 3 ( 28 ) : 0 ( 0 ) : 93 | 4.858 |
| 179 | SHANK2 | 3 ( 28 ) : 0 ( 0 ) : 94 | 4.858 |
| 180 | SLC4A4 | 0 ( 0 ) : 3 ( 28 ) : 95 | -4.858 |
| 181 | WDFY4 | 3 ( 28 ) : 0 ( 0 ) : 96 | 4.858 |
| 182 | LAMB1 | 3 ( 27 ) : 2 ( 27 ) : F : F | 0 |
| 183 | CD40LG | 3 ( 27 ) : 2 ( 21 ) : F : F | 0.3479 |
| 184 | SIMC1 | 2 ( 21 ) : 3 ( 27 ) : F : F | -0.3479 |
| 185 | ARID1A | 2 ( 17 ) : 3 ( 27 ) : F : F | -0.6374 |
| 186 | GPR176 | 2 ( 2 ) : 3 ( 26 ) : F : T | -3.1699 |
| 187 | ADAMTS12 | 1 ( 14 ) : 3 ( 26 ) : T : F | -0.848 |
| 188 | KIF6 | 1 ( 3 ) : 3 ( 26 ) : 97 | -2.7549 |
| 189 | ADGRD2 | 3 ( 26 ) : 0 ( 0 ) : 98 | 4.7549 |
| 190 | SLC12A4 | 3 ( 26 ) : 0 ( 0 ) : 99 | 4.7549 |
| 191 | DNER | 3 ( 25 ) : 3 ( 21 ) : F : F | 0.241 |
| 192 | BCL11A | 1 ( 25 ) : 3 ( 22 ) : T : F | 0.1769 |
| 193 | ABI3BP | 3 ( 25 ) : 1 ( 5 ) : 100 | 2.1155 |
| 194 | ZAN | 3 ( 25 ) : 1 ( 5 ) : 101 | 2.1155 |
| 195 | ABCA4 | 3 ( 25 ) : 0 ( 0 ) : 102 | 4.7004 |
| 196 | CNST | 0 ( 0 ) : 3 ( 25 ) : 103 | -4.7004 |
| 197 | KLHL4 | 0 ( 0 ) : 3 ( 25 ) : 104 | -4.7004 |
| 198 | ZNF717 | 3 ( 25 ) : 0 ( 0 ) : 105 | 4.7004 |
| 199 | ZNF638 | 3 ( 24 ) : 3 ( 16 ) : F : F | 0.5564 |
| 200 | LRP1B | 2 ( 17 ) : 3 ( 24 ) : F : F | -0.4739 |
| 201 | LRBA | 2 ( 12 ) : 3 ( 24 ) : F : F | -0.9434 |
| 202 | ITPR1 | 3 ( 24 ) : 1 ( 11 ) : 106 | 1.0589 |
| 203 | ULK4 | 3 ( 24 ) : 1 ( 3 ) : 107 | 2.6439 |
| 204 | NBPF10 | 1 ( 2 ) : 3 ( 24 ) : 108 | -3.0589 |
| 205 | KCNQ2 | 0 ( 0 ) : 3 ( 24 ) : 109 | -4.6439 |
| 206 | ZNF658 | 3 ( 24 ) : 0 ( 0 ) : 110 | 4.6439 |
| 207 | STAB1 | 2 ( 23 ) : 3 ( 16 ) : F : F | 0.4975 |
| 208 | SRCAP | 1 ( 5 ) : 3 ( 23 ) : 111 | -2 |
| 209 | ADAM33 | 3 ( 23 ) : 0 ( 0 ) : 112 | 4.585 |
| 210 | BIN3 | 0 ( 0 ) : 3 ( 23 ) : 113 | -4.585 |
| 211 | ADCY1 | 3 ( 22 ) : 1 ( 3 ) : 114 | 2.5236 |
| 212 | AMBN | 0 ( 0 ) : 3 ( 22 ) : 115 | -4.5236 |
| 213 | TENM1 | 3 ( 22 ) : 0 ( 0 ) : 116 | 4.5236 |
| 214 | ABCA12 | 1 ( 7 ) : 3 ( 21 ) : 117 | -1.4594 |
| 215 | ERVK-7 | 1 ( 7 ) : 3 ( 21 ) : 118 | -1.4594 |
| 216 | ZNF804A | 3 ( 21 ) : 1 ( 5 ) : 119 | 1.8745 |
| 217 | PLXNA1 | 0 ( 0 ) : 3 ( 21 ) : 120 | -4.4594 |
| 218 | TNRC18 | 0 ( 0 ) : 3 ( 21 ) : 121 | -4.4594 |
| 219 | ZNRF3 | 0 ( 0 ) : 3 ( 21 ) : 122 | -4.4594 |
| 220 | DST | 3 ( 20 ) : 1 ( 9 ) : 123 | 1.0704 |
| 221 | CIC | 0 ( 0 ) : 3 ( 20 ) : 124 | -4.3923 |
| 222 | OR8B8 | 3 ( 20 ) : 0 ( 0 ) : 125 | 4.3923 |
| 223 | PLXNA3 | 0 ( 0 ) : 3 ( 20 ) : 126 | -4.3923 |
| 224 | CAMTA1 | 1 ( 7 ) : 3 ( 19 ) : 127 | -1.3219 |
| 225 | PREX1 | 3 ( 19 ) : 1 ( 7 ) : 128 | 1.3219 |
| 226 | MUC19 | 3 ( 16 ) : 3 ( 18 ) : F : F | -0.1605 |
| 227 | NOTCH1 | 3 ( 16 ) : 3 ( 18 ) : F : F | -0.1605 |
| 228 | SETBP1 | 3 ( 18 ) : 1 ( 14 ) : T : F | 0.341 |
| 229 | ITGA3 | 1 ( 5 ) : 3 ( 18 ) : 129 | -1.663 |
| 230 | MC3R | 0 ( 0 ) : 3 ( 18 ) : 130 | -4.2479 |
| 231 | LINC01000 | 2 ( 14 ) : 3 ( 17 ) : F : F | -0.263 |
| 232 | KLK6 | 1 ( 11 ) : 3 ( 17 ) : T : F | -0.585 |
| 233 | TCF20 | 1 ( 5 ) : 3 ( 17 ) : 131 | -1.585 |
| 234 | PCDHB12 | 3 ( 17 ) : 1 ( 3 ) : 132 | 2.1699 |
| 235 | CARM1 | 0 ( 0 ) : 3 ( 17 ) : 133 | -4.1699 |
| 236 | HTR5A | 0 ( 0 ) : 3 ( 17 ) : 134 | -4.1699 |
| 237 | PCDHB7 | 3 ( 16 ) : 2 ( 10 ) : F : F | 0.628 |
| 238 | C2CD6 | 3 ( 16 ) : 0 ( 0 ) : 135 | 4.0875 |
| 239 | CCDC141 | 0 ( 0 ) : 3 ( 16 ) : 136 | -4.0875 |
| 240 | CRIM1 | 3 ( 15 ) : 1 ( 9 ) : T : F | 0.6781 |
| 241 | ATP10A | 3 ( 15 ) : 1 ( 4 ) : 137 | 1.6781 |
| 242 | FAT2 | 3 ( 15 ) : 1 ( 3 ) : 138 | 2 |
| 243 | FIGN | 3 ( 15 ) : 0 ( 0 ) : 139 | 4 |
| 244 | PPAN | 3 ( 15 ) : 0 ( 0 ) : 140 | 4 |
| 245 | TNN | 0 ( 0 ) : 3 ( 15 ) : 141 | -4 |
| 246 | CACNG8 | 0 ( 0 ) : 3 ( 14 ) : 142 | -3.9069 |
| 247 | RBM15B | 3 ( 14 ) : 0 ( 0 ) : 143 | 3.9069 |
| 248 | TGS1 | 3 ( 14 ) : 0 ( 0 ) : 144 | 3.9069 |
| 249 | UNC5A | 0 ( 0 ) : 3 ( 14 ) : 145 | -3.9069 |
| 250 | ZNF646 | 3 ( 14 ) : 0 ( 0 ) : 146 | 3.9069 |
| 251 | MAP1A | 2 ( 13 ) : 3 ( 13 ) : F : F | 0 |
| 252 | C20orf173 | 3 ( 13 ) : 1 ( 5 ) : 147 | 1.2224 |
| 253 | RTL9 | 1 ( 6 ) : 3 ( 12 ) : T : F | -0.8931 |
| 254 | HERC1 | 2 ( 9 ) : 3 ( 11 ) : F : F | -0.263 |
| 255 | PLXNA2 | 1 ( 7 ) : 3 ( 11 ) : T : F | -0.585 |
| 256 | FBN1 | 1 ( 2 ) : 3 ( 11 ) : 148 | -2 |
| 257 | C7orf43 | 3 ( 10 ) : 0 ( 0 ) : 149 | 3.4594 |
| 258 | IGHM | 2 ( 6 ) : 3 ( 9 ) : F : F | -0.5146 |
| 259 | EP300 | 3 ( 9 ) : 1 ( 3 ) : 150 | 1.3219 |
| 260 | CCDC93 | 0 ( 0 ) : 3 ( 9 ) : 151 | -3.3219 |
| 261 | SLC33A1 | 0 ( 0 ) : 3 ( 9 ) : 152 | -3.3219 |
| 262 | AMER1 | 0 ( 0 ) : 3 ( 8 ) : 153 | -3.1699 |
| 263 | CAPN15 | 3 ( 6 ) : 0 ( 0 ) : 154 | 2.8074 |
