# Supplementary material for: Distinguishing patients with idiopathic epilepsy from solitary cysticercus granuloma epilepsy and biochemical phenotype assessment using a serum biomolecule profiling platform
Source: PLoS One. 2020 Aug 21;15(8):e0237064. doi: 10.1371/journal.pone.0237064 (PMC7527271; doi:10.1371/journal.pone.0237064)
Supplement: S2 Table — (DOCX) [file pone.0237064.s005.docx]

**S3 Table: Patients used for each figure**

| Figure and Panel | order in figure | Group | Coded Patient Number | Group Pathology | Gender | Age |
| --- | --- | --- | --- | --- | --- | --- |
| Fig 2 Panel A | 1 | SCG, N=29 | Patient_34 | SCG | F | 18 |
| Fig 2 Panel A | 2 | SCG, N=29 | Patient_31 | SCG | F | 20 |
| Fig 2 Panel A | 3 | SCG, N=29 | Patient_35 | SCG | F | 29 |
| Fig 2 Panel A | 4 | SCG, N=29 | Patient_36 | SCG | F | 31 |
| Fig 2 Panel A | 5 | SCG, N=29 | Patient_37 | SCG | F | 48 |
| Fig 2 Panel A | 6 | SCG, N=29 | Patient_55 | SCG | M | 18 |
| Fig 2 Panel A | 7 | SCG, N=29 | Patient_38 | SCG | M | 18 |
| Fig 2 Panel A | 8 | SCG, N=29 | Patient_39 | SCG | M | 18 |
| Fig 2 Panel A | 9 | SCG, N=29 | Patient_40 | SCG | M | 19 |
| Fig 2 Panel A | 10 | SCG, N=29 | Patient_41 | SCG | M | 20 |
| Fig 2 Panel A | 11 | SCG, N=29 | Patient_30 | SCG | M | 21 |
| Fig 2 Panel A | 12 | SCG, N=29 | Patient_56 | SCG | M | 23 |
| Fig 2 Panel A | 13 | SCG, N=29 | Patient_42 | SCG | M | 23 |
| Fig 2 Panel A | 14 | SCG, N=29 | Patient_57 | SCG | M | 24 |
| Fig 2 Panel A | 15 | SCG, N=29 | Patient_43 | SCG | M | 26 |
| Fig 2 Panel A | 16 | SCG, N=29 | Patient_44 | SCG | M | 26 |
| Fig 2 Panel A | 17 | SCG, N=29 | Patient_45 | SCG | M | 28 |
| Fig 2 Panel A | 18 | SCG, N=29 | Patient_46 | SCG | M | 29 |
| Fig 2 Panel A | 19 | SCG, N=29 | Patient_33 | SCG | M | 30 |
| Fig 2 Panel A | 20 | SCG, N=29 | Patient_47 | SCG | M | 33 |
| Fig 2 Panel A | 21 | SCG, N=29 | Patient_48 | SCG | M | 35 |
| Fig 2 Panel A | 22 | SCG, N=29 | Patient_49 | SCG | M | 39 |
| Fig 2 Panel A | 23 | SCG, N=29 | Patient_58 | SCG | M | 40 |
| Fig 2 Panel A | 24 | SCG, N=29 | Patient_50 | SCG | M | 42 |
| Fig 2 Panel A | 25 | SCG, N=29 | Patient_51 | SCG | M | 42 |
| Fig 2 Panel A | 26 | SCG, N=29 | Patient_32 | SCG | M | 42 |
| Fig 2 Panel A | 27 | SCG, N=29 | Patient_52 | SCG | M | 45 |
| Fig 2 Panel A | 28 | SCG, N=29 | Patient_53 | SCG | M | 48 |
| Fig 2 Panel A | 29 | SCG, N=29 | Patient_54 | SCG | M | 48 |
| Fig 2 Panel A | 30 | IE, N=29 | Patient_01 | IE | F | 19 |
| Fig 2 Panel A | 31 | IE, N=29 | Patient_02 | IE | F | 19 |
| Fig 2 Panel A | 32 | IE, N=29 | Patient_03 | IE | F | 21 |
| Fig 2 Panel A | 33 | IE, N=29 | Patient_29 | IE | F | 22 |
| Fig 2 Panel A | 34 | IE, N=29 | Patient_04 | IE | F | 23 |
| Fig 2 Panel A | 35 | IE, N=29 | Patient_05 | IE | F | 28 |
| Fig 2 Panel A | 36 | IE, N=29 | Patient_06 | IE | F | 30 |
| Fig 2 Panel A | 37 | IE, N=29 | Patient_07 | IE | F | 32 |
| Fig 2 Panel A | 38 | IE, N=29 | Patient_22 | IE | F | 34 |
| Fig 2 Panel A | 39 | IE, N=29 | Patient_28 | IE | F | 40 |
| Fig 2 Panel A | 40 | IE, N=29 | Patient_08 | IE | M | 19 |
| Fig 2 Panel A | 41 | IE, N=29 | Patient_09 | IE | M | 19 |
| Fig 2 Panel A | 42 | IE, N=29 | Patient_10 | IE | M | 20 |
| Fig 2 Panel A | 43 | IE, N=29 | Patient_11 | IE | M | 20 |
| Fig 2 Panel A | 44 | IE, N=29 | Patient_12 | IE | M | 21 |
| Fig 2 Panel A | 45 | IE, N=29 | Patient_23 | IE | M | 22 |
| Fig 2 Panel A | 46 | IE, N=29 | Patient_13 | IE | M | 22 |
| Fig 2 Panel A | 47 | IE, N=29 | Patient_24 | IE | M | 23 |
| Fig 2 Panel A | 48 | IE, N=29 | Patient_14 | IE | M | 24 |
| Fig 2 Panel A | 49 | IE, N=29 | Patient_15 | IE | M | 24 |
| Fig 2 Panel A | 50 | IE, N=29 | Patient_27 | IE | M | 26 |
| Fig 2 Panel A | 51 | IE, N=29 | Patient_16 | IE | M | 27 |
| Fig 2 Panel A | 52 | IE, N=29 | Patient_25 | IE | M | 30 |
| Fig 2 Panel A | 53 | IE, N=29 | Patient_17 | IE | M | 31 |
| Fig 2 Panel A | 54 | IE, N=29 | Patient_18 | IE | M | 33 |
| Fig 2 Panel A | 55 | IE, N=29 | Patient_26 | IE | M | 33 |
| Fig 2 Panel A | 56 | IE, N=29 | Patient_19 | IE | M | 33 |
| Fig 2 Panel A | 57 | IE, N=29 | Patient_20 | IE | M | 40 |
| Fig 2 Panel A | 58 | IE, N=29 | Patient_21 | IE | M | 46 |
| Fig 2 Panel B | 59 | RND SCG, N=29 | Patient_34 | SCG | F | 18 |
| Fig 2 Panel B | 60 | RND SCG, N=29 | Patient_35 | SCG | F | 29 |
| Fig 2 Panel B | 61 | RND SCG, N=29 | Patient_37 | SCG | F | 48 |
| Fig 2 Panel B | 62 | RND SCG, N=29 | Patient_55 | SCG | M | 18 |
| Fig 2 Panel B | 63 | RND SCG, N=29 | Patient_39 | SCG | M | 18 |
| Fig 2 Panel B | 64 | RND SCG, N=29 | Patient_41 | SCG | M | 20 |
| Fig 2 Panel B | 65 | RND SCG, N=29 | Patient_56 | SCG | M | 23 |
| Fig 2 Panel B | 66 | RND SCG, N=29 | Patient_57 | SCG | M | 24 |
| Fig 2 Panel B | 67 | RND SCG, N=29 | Patient_44 | SCG | M | 26 |
| Fig 2 Panel B | 68 | RND SCG, N=29 | Patient_46 | SCG | M | 29 |
| Fig 2 Panel B | 69 | RND SCG, N=29 | Patient_47 | SCG | M | 33 |
| Fig 2 Panel B | 70 | RND SCG, N=29 | Patient_49 | SCG | M | 39 |
| Fig 2 Panel B | 71 | RND SCG, N=29 | Patient_50 | SCG | M | 42 |
| Fig 2 Panel B | 72 | RND SCG, N=29 | Patient_32 | SCG | M | 42 |
| Fig 2 Panel B | 73 | RND SCG, N=29 | Patient_53 | SCG | M | 48 |
| Fig 2 Panel B | 74 | RND SCG, N=29 | Patient_54 | SCG | M | 48 |
| Fig 2 Panel B | 75 | RND SCG, N=29 | Patient_02 | IE | F | 19 |
| Fig 2 Panel B | 76 | RND SCG, N=29 | Patient_29 | IE | F | 22 |
| Fig 2 Panel B | 77 | RND SCG, N=29 | Patient_08 | IE | M | 19 |
| Fig 2 Panel B | 78 | RND SCG, N=29 | Patient_10 | IE | M | 20 |
| Fig 2 Panel B | 79 | RND SCG, N=29 | Patient_12 | IE | M | 21 |
| Fig 2 Panel B | 80 | RND SCG, N=29 | Patient_13 | IE | M | 22 |
| Fig 2 Panel B | 81 | RND SCG, N=29 | Patient_14 | IE | M | 24 |
| Fig 2 Panel B | 82 | RND SCG, N=29 | Patient_27 | IE | M | 26 |
| Fig 2 Panel B | 83 | RND SCG, N=29 | Patient_25 | IE | M | 30 |
| Fig 2 Panel B | 84 | RND SCG, N=29 | Patient_18 | IE | M | 33 |
| Fig 2 Panel B | 85 | RND SCG, N=29 | Patient_19 | IE | M | 33 |
| Fig 2 Panel B | 86 | RND SCG, N=29 | Patient_20 | IE | M | 40 |
| Fig 2 Panel B | 87 | RND SCG, N=29 | Patient_21 | IE | M | 46 |
| Fig 2 Panel B | 88 | RND IE, N=29 | Patient_31 | SCG | F | 20 |
| Fig 2 Panel B | 89 | RND IE, N=29 | Patient_36 | SCG | F | 31 |
| Fig 2 Panel B | 90 | RND IE, N=29 | Patient_38 | SCG | M | 18 |
| Fig 2 Panel B | 91 | RND IE, N=29 | Patient_40 | SCG | M | 19 |
| Fig 2 Panel B | 92 | RND IE, N=29 | Patient_30 | SCG | M | 21 |
| Fig 2 Panel B | 93 | RND IE, N=29 | Patient_42 | SCG | M | 23 |
| Fig 2 Panel B | 94 | RND IE, N=29 | Patient_43 | SCG | M | 26 |
| Fig 2 Panel B | 95 | RND IE, N=29 | Patient_45 | SCG | M | 28 |
| Fig 2 Panel B | 96 | RND IE, N=29 | Patient_33 | SCG | M | 30 |
| Fig 2 Panel B | 97 | RND IE, N=29 | Patient_48 | SCG | M | 35 |
| Fig 2 Panel B | 98 | RND IE, N=29 | Patient_58 | SCG | M | 40 |
| Fig 2 Panel B | 99 | RND IE, N=29 | Patient_51 | SCG | M | 42 |
| Fig 2 Panel B | 100 | RND IE, N=29 | Patient_52 | SCG | M | 45 |
| Fig 2 Panel B | 101 | RND IE, N=29 | Patient_01 | IE | F | 19 |
| Fig 2 Panel B | 102 | RND IE, N=29 | Patient_03 | IE | F | 21 |
| Fig 2 Panel B | 103 | RND IE, N=29 | Patient_04 | IE | F | 23 |
| Fig 2 Panel B | 104 | RND IE, N=29 | Patient_05 | IE | F | 28 |
| Fig 2 Panel B | 105 | RND IE, N=29 | Patient_06 | IE | F | 30 |
| Fig 2 Panel B | 106 | RND IE, N=29 | Patient_07 | IE | F | 32 |
| Fig 2 Panel B | 107 | RND IE, N=29 | Patient_22 | IE | F | 34 |
| Fig 2 Panel B | 108 | RND IE, N=29 | Patient_28 | IE | F | 40 |
| Fig 2 Panel B | 109 | RND IE, N=29 | Patient_09 | IE | M | 19 |
| Fig 2 Panel B | 110 | RND IE, N=29 | Patient_11 | IE | M | 20 |
| Fig 2 Panel B | 111 | RND IE, N=29 | Patient_23 | IE | M | 22 |
| Fig 2 Panel B | 112 | RND IE, N=29 | Patient_24 | IE | M | 23 |
| Fig 2 Panel B | 113 | RND IE, N=29 | Patient_15 | IE | M | 24 |
| Fig 2 Panel B | 114 | RND IE, N=29 | Patient_16 | IE | M | 27 |
| Fig 2 Panel B | 115 | RND IE, N=29 | Patient_17 | IE | M | 31 |
| Fig 2 Panel B | 116 | RND IE, N=29 | Patient_26 | IE | M | 33 |
| S1 Fig Panel A | 1 | IE, N=29 | Patient_01 | IE | F | 19 |
| S1 Fig Panel A | 2 | IE, N=29 | Patient_02 | IE | F | 19 |
| S1 Fig Panel A | 3 | IE, N=29 | Patient_03 | IE | F | 21 |
| S1 Fig Panel A | 4 | IE, N=29 | Patient_29 | IE | F | 22 |
| S1 Fig Panel A | 5 | IE, N=29 | Patient_04 | IE | F | 23 |
| S1 Fig Panel A | 6 | IE, N=29 | Patient_05 | IE | F | 28 |
| S1 Fig Panel A | 7 | IE, N=29 | Patient_06 | IE | F | 30 |
| S1 Fig Panel A | 8 | IE, N=29 | Patient_07 | IE | F | 32 |
| S1 Fig Panel A | 9 | IE, N=29 | Patient_22 | IE | F | 34 |
| S1 Fig Panel A | 10 | IE, N=29 | Patient_28 | IE | F | 40 |
| S1 Fig Panel A | 11 | IE, N=29 | Patient_08 | IE | M | 19 |
| S1 Fig Panel A | 12 | IE, N=29 | Patient_09 | IE | M | 19 |
| S1 Fig Panel A | 13 | IE, N=29 | Patient_10 | IE | M | 20 |
| S1 Fig Panel A | 14 | IE, N=29 | Patient_11 | IE | M | 20 |
| S1 Fig Panel A | 15 | IE, N=29 | Patient_12 | IE | M | 21 |
| S1 Fig Panel A | 16 | IE, N=29 | Patient_23 | IE | M | 22 |
| S1 Fig Panel A | 17 | IE, N=29 | Patient_13 | IE | M | 22 |
| S1 Fig Panel A | 18 | IE, N=29 | Patient_24 | IE | M | 23 |
| S1 Fig Panel A | 19 | IE, N=29 | Patient_14 | IE | M | 24 |
| S1 Fig Panel A | 20 | IE, N=29 | Patient_15 | IE | M | 24 |
| S1 Fig Panel A | 21 | IE, N=29 | Patient_27 | IE | M | 26 |
| S1 Fig Panel A | 22 | IE, N=29 | Patient_16 | IE | M | 27 |
| S1 Fig Panel A | 23 | IE, N=29 | Patient_25 | IE | M | 30 |
| S1 Fig Panel A | 24 | IE, N=29 | Patient_17 | IE | M | 31 |
| S1 Fig Panel A | 25 | IE, N=29 | Patient_18 | IE | M | 33 |
| S1 Fig Panel A | 26 | IE, N=29 | Patient_26 | IE | M | 33 |
| S1 Fig Panel A | 27 | IE, N=29 | Patient_19 | IE | M | 33 |
| S1 Fig Panel A | 28 | IE, N=29 | Patient_20 | IE | M | 40 |
| S1 Fig Panel A | 29 | IE, N=29 | Patient_21 | IE | M | 46 |
| S1 Fig Panel A | 30 | SCG, N=29 | Patient_34 | SCG | F | 18 |
| S1 Fig Panel A | 31 | SCG, N=29 | Patient_31 | SCG | F | 20 |
| S1 Fig Panel A | 32 | SCG, N=29 | Patient_35 | SCG | F | 29 |
| S1 Fig Panel A | 33 | SCG, N=29 | Patient_36 | SCG | F | 31 |
| S1 Fig Panel A | 34 | SCG, N=29 | Patient_37 | SCG | F | 48 |
| S1 Fig Panel A | 35 | SCG, N=29 | Patient_55 | SCG | M | 18 |
| S1 Fig Panel A | 36 | SCG, N=29 | Patient_38 | SCG | M | 18 |
| S1 Fig Panel A | 37 | SCG, N=29 | Patient_39 | SCG | M | 18 |
| S1 Fig Panel A | 38 | SCG, N=29 | Patient_40 | SCG | M | 19 |
| S1 Fig Panel A | 39 | SCG, N=29 | Patient_41 | SCG | M | 20 |
| S1 Fig Panel A | 40 | SCG, N=29 | Patient_30 | SCG | M | 21 |
| S1 Fig Panel A | 41 | SCG, N=29 | Patient_56 | SCG | M | 23 |
| S1 Fig Panel A | 42 | SCG, N=29 | Patient_42 | SCG | M | 23 |
| S1 Fig Panel A | 43 | SCG, N=29 | Patient_57 | SCG | M | 24 |
| S1 Fig Panel A | 44 | SCG, N=29 | Patient_43 | SCG | M | 26 |
| S1 Fig Panel A | 45 | SCG, N=29 | Patient_44 | SCG | M | 26 |
| S1 Fig Panel A | 46 | SCG, N=29 | Patient_45 | SCG | M | 28 |
| S1 Fig Panel A | 47 | SCG, N=29 | Patient_46 | SCG | M | 29 |
| S1 Fig Panel A | 48 | SCG, N=29 | Patient_33 | SCG | M | 30 |
| S1 Fig Panel A | 49 | SCG, N=29 | Patient_47 | SCG | M | 33 |
| S1 Fig Panel A | 50 | SCG, N=29 | Patient_48 | SCG | M | 35 |
| S1 Fig Panel A | 51 | SCG, N=29 | Patient_49 | SCG | M | 39 |
| S1 Fig Panel A | 52 | SCG, N=29 | Patient_58 | SCG | M | 40 |
| S1 Fig Panel A | 53 | SCG, N=29 | Patient_50 | SCG | M | 42 |
| S1 Fig Panel A | 54 | SCG, N=29 | Patient_51 | SCG | M | 42 |
| S1 Fig Panel A | 55 | SCG, N=29 | Patient_32 | SCG | M | 42 |
| S1 Fig Panel A | 56 | SCG, N=29 | Patient_52 | SCG | M | 45 |
| S1 Fig Panel A | 57 | SCG, N=29 | Patient_53 | SCG | M | 48 |
| S1 Fig Panel A | 58 | SCG, N=29 | Patient_54 | SCG | M | 48 |
| S1 Fig Panel B | 59 | RND IE, N=29 | Patient_01 | IE | F | 19 |
| S1 Fig Panel B | 60 | RND IE, N=29 | Patient_03 | IE | F | 21 |
| S1 Fig Panel B | 61 | RND IE, N=29 | Patient_04 | IE | F | 23 |
| S1 Fig Panel B | 62 | RND IE, N=29 | Patient_06 | IE | F | 30 |
| S1 Fig Panel B | 63 | RND IE, N=29 | Patient_22 | IE | F | 34 |
| S1 Fig Panel B | 64 | RND IE, N=29 | Patient_08 | IE | M | 19 |
| S1 Fig Panel B | 65 | RND IE, N=29 | Patient_10 | IE | M | 20 |
| S1 Fig Panel B | 66 | RND IE, N=29 | Patient_12 | IE | M | 21 |
| S1 Fig Panel B | 67 | RND IE, N=29 | Patient_13 | IE | M | 22 |
| S1 Fig Panel B | 68 | RND IE, N=29 | Patient_14 | IE | M | 24 |
| S1 Fig Panel B | 69 | RND IE, N=29 | Patient_27 | IE | M | 26 |
| S1 Fig Panel B | 70 | RND IE, N=29 | Patient_25 | IE | M | 30 |
| S1 Fig Panel B | 71 | RND IE, N=29 | Patient_18 | IE | M | 33 |
| S1 Fig Panel B | 72 | RND IE, N=29 | Patient_19 | IE | M | 33 |
| S1 Fig Panel B | 73 | RND IE, N=29 | Patient_21 | IE | M | 46 |
| S1 Fig Panel B | 74 | RND IE, N=29 | Patient_31 | SCG | F | 20 |
| S1 Fig Panel B | 75 | RND IE, N=29 | Patient_36 | SCG | F | 31 |
| S1 Fig Panel B | 76 | RND IE, N=29 | Patient_55 | SCG | M | 18 |
| S1 Fig Panel B | 77 | RND IE, N=29 | Patient_39 | SCG | M | 18 |
| S1 Fig Panel B | 78 | RND IE, N=29 | Patient_41 | SCG | M | 20 |
| S1 Fig Panel B | 79 | RND IE, N=29 | Patient_56 | SCG | M | 23 |
| S1 Fig Panel B | 80 | RND IE, N=29 | Patient_57 | SCG | M | 24 |
| S1 Fig Panel B | 81 | RND IE, N=29 | Patient_44 | SCG | M | 26 |
| S1 Fig Panel B | 82 | RND IE, N=29 | Patient_46 | SCG | M | 29 |
| S1 Fig Panel B | 83 | RND IE, N=29 | Patient_47 | SCG | M | 33 |
| S1 Fig Panel B | 84 | RND IE, N=29 | Patient_49 | SCG | M | 39 |
| S1 Fig Panel B | 85 | RND IE, N=29 | Patient_50 | SCG | M | 42 |
| S1 Fig Panel B | 86 | RND IE, N=29 | Patient_32 | SCG | M | 42 |
| S1 Fig Panel B | 87 | RND IE, N=29 | Patient_53 | SCG | M | 48 |
| S1 Fig Panel B | 88 | RND SCG, N=29 | Patient_02 | IE | F | 19 |
| S1 Fig Panel B | 89 | RND SCG, N=29 | Patient_29 | IE | F | 22 |
| S1 Fig Panel B | 90 | RND SCG, N=29 | Patient_05 | IE | F | 28 |
| S1 Fig Panel B | 91 | RND SCG, N=29 | Patient_07 | IE | F | 32 |
| S1 Fig Panel B | 92 | RND SCG, N=29 | Patient_28 | IE | F | 40 |
| S1 Fig Panel B | 93 | RND SCG, N=29 | Patient_09 | IE | M | 19 |
| S1 Fig Panel B | 94 | RND SCG, N=29 | Patient_11 | IE | M | 20 |
| S1 Fig Panel B | 95 | RND SCG, N=29 | Patient_23 | IE | M | 22 |
| S1 Fig Panel B | 96 | RND SCG, N=29 | Patient_24 | IE | M | 23 |
| S1 Fig Panel B | 97 | RND SCG, N=29 | Patient_15 | IE | M | 24 |
| S1 Fig Panel B | 98 | RND SCG, N=29 | Patient_16 | IE | M | 27 |
| S1 Fig Panel B | 99 | RND SCG, N=29 | Patient_17 | IE | M | 31 |
| S1 Fig Panel B | 100 | RND SCG, N=29 | Patient_26 | IE | M | 33 |
| S1 Fig Panel B | 101 | RND SCG, N=29 | Patient_20 | IE | M | 40 |
| S1 Fig Panel B | 102 | RND SCG, N=29 | Patient_34 | SCG | F | 18 |
| S1 Fig Panel B | 103 | RND SCG, N=29 | Patient_35 | SCG | F | 29 |
| S1 Fig Panel B | 104 | RND SCG, N=29 | Patient_37 | SCG | F | 48 |
| S1 Fig Panel B | 105 | RND SCG, N=29 | Patient_38 | SCG | M | 18 |
| S1 Fig Panel B | 106 | RND SCG, N=29 | Patient_40 | SCG | M | 19 |
| S1 Fig Panel B | 107 | RND SCG, N=29 | Patient_30 | SCG | M | 21 |
| S1 Fig Panel B | 108 | RND SCG, N=29 | Patient_42 | SCG | M | 23 |
| S1 Fig Panel B | 109 | RND SCG, N=29 | Patient_43 | SCG | M | 26 |
| S1 Fig Panel B | 110 | RND SCG, N=29 | Patient_45 | SCG | M | 28 |
| S1 Fig Panel B | 111 | RND SCG, N=29 | Patient_33 | SCG | M | 30 |
| S1 Fig Panel B | 112 | RND SCG, N=29 | Patient_48 | SCG | M | 35 |
| S1 Fig Panel B | 113 | RND SCG, N=29 | Patient_58 | SCG | M | 40 |
| S1 Fig Panel B | 114 | RND SCG, N=29 | Patient_51 | SCG | M | 42 |
| S1 Fig Panel B | 115 | RND SCG, N=29 | Patient_52 | SCG | M | 45 |
| S1 Fig Panel B | 116 | RND SCG, N=29 | Patient_54 | SCG | M | 48 |
| S1 Fig Panel C | 1 | IE, N=25 | Patient_01 | IE | F | 19 |
| S1 Fig Panel C | 2 | IE, N=25 | Patient_02 | IE | F | 19 |
| S1 Fig Panel C | 3 | IE, N=25 | Patient_03 | IE | F | 21 |
| S1 Fig Panel C | 4 | IE, N=25 | Patient_04 | IE | F | 23 |
| S1 Fig Panel C | 5 | IE, N=25 | Patient_05 | IE | F | 28 |
| S1 Fig Panel C | 6 | IE, N=25 | Patient_06 | IE | F | 30 |
| S1 Fig Panel C | 7 | IE, N=25 | Patient_07 | IE | F | 32 |
| S1 Fig Panel C | 8 | IE, N=25 | Patient_08 | IE | M | 19 |
| S1 Fig Panel C | 9 | IE, N=25 | Patient_09 | IE | M | 19 |
| S1 Fig Panel C | 10 | IE, N=25 | Patient_10 | IE | M | 20 |
| S1 Fig Panel C | 11 | IE, N=25 | Patient_11 | IE | M | 20 |
| S1 Fig Panel C | 12 | IE, N=25 | Patient_12 | IE | M | 21 |
| S1 Fig Panel C | 13 | IE, N=25 | Patient_13 | IE | M | 22 |
| S1 Fig Panel C | 14 | IE, N=25 | Patient_14 | IE | M | 24 |
| S1 Fig Panel C | 15 | IE, N=25 | Patient_15 | IE | M | 24 |
| S1 Fig Panel C | 16 | IE, N=25 | Patient_16 | IE | M | 27 |
| S1 Fig Panel C | 17 | IE, N=25 | Patient_17 | IE | M | 31 |
| S1 Fig Panel C | 18 | IE, N=25 | Patient_18 | IE | M | 33 |
| S1 Fig Panel C | 19 | IE, N=25 | Patient_19 | IE | M | 33 |
| S1 Fig Panel C | 20 | IE, N=25 | Patient_20 | IE | M | 40 |
| S1 Fig Panel C | 21 | IE, N=25 | Patient_21 | IE | M | 46 |
| S1 Fig Panel C | 22 | IE, N=25 | Patient_22 | IE | F | 34 |
| S1 Fig Panel C | 23 | IE, N=25 | Patient_23 | IE | M | 22 |
| S1 Fig Panel C | 24 | IE, N=25 | Patient_24 | IE | M | 23 |
| S1 Fig Panel C | 25 | IE, N=25 | Patient_25 | IE | M | 30 |
| S1 Fig Panel C | 26 | SCG, N=25 | Patient_34 | SCG | F | 18 |
| S1 Fig Panel C | 27 | SCG, N=25 | Patient_35 | SCG | F | 29 |
| S1 Fig Panel C | 28 | SCG, N=25 | Patient_36 | SCG | F | 31 |
| S1 Fig Panel C | 29 | SCG, N=25 | Patient_37 | SCG | F | 48 |
| S1 Fig Panel C | 30 | SCG, N=25 | Patient_38 | SCG | M | 18 |
| S1 Fig Panel C | 31 | SCG, N=25 | Patient_39 | SCG | M | 18 |
| S1 Fig Panel C | 32 | SCG, N=25 | Patient_40 | SCG | M | 19 |
| S1 Fig Panel C | 33 | SCG, N=25 | Patient_41 | SCG | M | 20 |
| S1 Fig Panel C | 34 | SCG, N=25 | Patient_42 | SCG | M | 23 |
| S1 Fig Panel C | 35 | SCG, N=25 | Patient_43 | SCG | M | 26 |
| S1 Fig Panel C | 36 | SCG, N=25 | Patient_44 | SCG | M | 26 |
| S1 Fig Panel C | 37 | SCG, N=25 | Patient_45 | SCG | M | 28 |
| S1 Fig Panel C | 38 | SCG, N=25 | Patient_46 | SCG | M | 29 |
| S1 Fig Panel C | 39 | SCG, N=25 | Patient_47 | SCG | M | 33 |
| S1 Fig Panel C | 40 | SCG, N=25 | Patient_48 | SCG | M | 35 |
| S1 Fig Panel C | 41 | SCG, N=25 | Patient_49 | SCG | M | 39 |
| S1 Fig Panel C | 42 | SCG, N=25 | Patient_50 | SCG | M | 42 |
| S1 Fig Panel C | 43 | SCG, N=25 | Patient_51 | SCG | M | 42 |
| S1 Fig Panel C | 44 | SCG, N=25 | Patient_52 | SCG | M | 45 |
| S1 Fig Panel C | 45 | SCG, N=25 | Patient_53 | SCG | M | 48 |
| S1 Fig Panel C | 46 | SCG, N=25 | Patient_54 | SCG | M | 48 |
| S1 Fig Panel C | 47 | SCG, N=25 | Patient_55 | SCG | M | 18 |
| S1 Fig Panel C | 48 | SCG, N=25 | Patient_56 | SCG | M | 23 |
| S1 Fig Panel C | 49 | SCG, N=25 | Patient_57 | SCG | M | 24 |
| S1 Fig Panel C | 50 | SCG, N=25 | Patient_58 | SCG | M | 40 |
| S1 Fig Panel C | 51 | RND IE, N=25 | Patient_01 | IE | F | 19 |
| S1 Fig Panel C | 52 | RND IE, N=25 | Patient_03 | IE | F | 21 |
| S1 Fig Panel C | 53 | RND IE, N=25 | Patient_05 | IE | F | 28 |
| S1 Fig Panel C | 54 | RND IE, N=25 | Patient_07 | IE | F | 32 |
| S1 Fig Panel C | 55 | RND IE, N=25 | Patient_09 | IE | M | 19 |
| S1 Fig Panel C | 56 | RND IE, N=25 | Patient_11 | IE | M | 20 |
| S1 Fig Panel C | 57 | RND IE, N=25 | Patient_13 | IE | M | 22 |
| S1 Fig Panel C | 58 | RND IE, N=25 | Patient_15 | IE | M | 24 |
| S1 Fig Panel C | 59 | RND IE, N=25 | Patient_17 | IE | M | 31 |
| S1 Fig Panel C | 60 | RND IE, N=25 | Patient_19 | IE | M | 33 |
| S1 Fig Panel C | 61 | RND IE, N=25 | Patient_21 | IE | M | 46 |
| S1 Fig Panel C | 62 | RND IE, N=25 | Patient_23 | IE | M | 22 |
| S1 Fig Panel C | 63 | RND IE, N=25 | Patient_25 | IE | M | 30 |
| S1 Fig Panel C | 64 | RND IE, N=25 | Patient_35 | SCG | F | 29 |
| S1 Fig Panel C | 65 | RND IE, N=25 | Patient_37 | SCG | F | 48 |
| S1 Fig Panel C | 66 | RND IE, N=25 | Patient_39 | SCG | M | 18 |
| S1 Fig Panel C | 67 | RND IE, N=25 | Patient_41 | SCG | M | 20 |
| S1 Fig Panel C | 68 | RND IE, N=25 | Patient_43 | SCG | M | 26 |
| S1 Fig Panel C | 69 | RND IE, N=25 | Patient_45 | SCG | M | 28 |
| S1 Fig Panel C | 70 | RND IE, N=25 | Patient_47 | SCG | M | 33 |
| S1 Fig Panel C | 71 | RND IE, N=25 | Patient_49 | SCG | M | 39 |
| S1 Fig Panel C | 72 | RND IE, N=25 | Patient_51 | SCG | M | 42 |
| S1 Fig Panel C | 73 | RND IE, N=25 | Patient_53 | SCG | M | 48 |
| S1 Fig Panel C | 74 | RND IE, N=25 | Patient_55 | SCG | M | 18 |
| S1 Fig Panel C | 75 | RND IE, N=25 | Patient_57 | SCG | M | 24 |
| S1 Fig Panel C | 76 | RND SCG, N=25 | Patient_02 | IE | F | 19 |
| S1 Fig Panel C | 77 | RND SCG, N=25 | Patient_04 | IE | F | 23 |
| S1 Fig Panel C | 78 | RND SCG, N=25 | Patient_06 | IE | F | 30 |
| S1 Fig Panel C | 79 | RND SCG, N=25 | Patient_08 | IE | M | 19 |
| S1 Fig Panel C | 80 | RND SCG, N=25 | Patient_10 | IE | M | 20 |
| S1 Fig Panel C | 81 | RND SCG, N=25 | Patient_12 | IE | M | 21 |
| S1 Fig Panel C | 82 | RND SCG, N=25 | Patient_14 | IE | M | 24 |
| S1 Fig Panel C | 83 | RND SCG, N=25 | Patient_16 | IE | M | 27 |
| S1 Fig Panel C | 84 | RND SCG, N=25 | Patient_18 | IE | M | 33 |
| S1 Fig Panel C | 85 | RND SCG, N=25 | Patient_20 | IE | M | 40 |
| S1 Fig Panel C | 86 | RND SCG, N=25 | Patient_22 | IE | F | 34 |
| S1 Fig Panel C | 87 | RND SCG, N=25 | Patient_24 | IE | M | 23 |
| S1 Fig Panel C | 88 | RND SCG, N=25 | Patient_34 | SCG | F | 18 |
| S1 Fig Panel C | 89 | RND SCG, N=25 | Patient_36 | SCG | F | 31 |
| S1 Fig Panel C | 90 | RND SCG, N=25 | Patient_38 | SCG | M | 18 |
| S1 Fig Panel C | 91 | RND SCG, N=25 | Patient_40 | SCG | M | 19 |
| S1 Fig Panel C | 92 | RND SCG, N=25 | Patient_42 | SCG | M | 23 |
| S1 Fig Panel C | 93 | RND SCG, N=25 | Patient_44 | SCG | M | 26 |
| S1 Fig Panel C | 94 | RND SCG, N=25 | Patient_46 | SCG | M | 29 |
| S1 Fig Panel C | 95 | RND SCG, N=25 | Patient_48 | SCG | M | 35 |
| S1 Fig Panel C | 96 | RND SCG, N=25 | Patient_50 | SCG | M | 42 |
| S1 Fig Panel C | 97 | RND SCG, N=25 | Patient_52 | SCG | M | 45 |
| S1 Fig Panel C | 98 | RND SCG, N=25 | Patient_54 | SCG | M | 48 |
| S1 Fig Panel C | 99 | RND SCG, N=25 | Patient_56 | SCG | M | 23 |
| S1 Fig Panel C | 100 | RND SCG, N=25 | Patient_58 | SCG | M | 40 |
| S1 Fig Panel D | 1 | BLIND SCG N=4 | Patient_30 | SCG | M | 21 |
| S1 Fig Panel D | 2 | BLIND SCG N=4 | Patient_31 | SCG | F | 20 |
| S1 Fig Panel D | 3 | BLIND SCG N=4 | Patient_32 | SCG | M | 42 |
| S1 Fig Panel D | 4 | BLIND SCG N=4 | Patient_33 | SCG | M | 30 |
| S1 Fig Panel D | 5 | BLIND IE, N=4 | Patient_26 | IE | M | 33 |
| S1 Fig Panel D | 6 | BLIND IE, N=4 | Patient_27 | IE | M | 26 |
| S1 Fig Panel D | 7 | BLIND IE, N=4 | Patient_28 | IE | F | 40 |
| S1 Fig Panel D | 8 | BLIND IE, N=4 | Patient_29 | IE | F | 22 |
| S3 Fig Panel A | 1 | IE, N=22 | Patient_01 | IE | F | 19 |
| S3 Fig Panel A | 2 | IE, N=22 | Patient_02 | IE | F | 19 |
| S3 Fig Panel A | 3 | IE, N=22 | Patient_03 | IE | F | 21 |
| S3 Fig Panel A | 4 | IE, N=22 | Patient_29 | IE | F | 22 |
| S3 Fig Panel A | 5 | IE, N=22 | Patient_04 | IE | F | 23 |
| S3 Fig Panel A | 6 | IE, N=22 | Patient_05 | IE | F | 28 |
| S3 Fig Panel A | 7 | IE, N=22 | Patient_06 | IE | F | 30 |
| S3 Fig Panel A | 8 | IE, N=22 | Patient_07 | IE | F | 32 |
| S3 Fig Panel A | 9 | IE, N=22 | Patient_28 | IE | F | 40 |
| S3 Fig Panel A | 10 | IE, N=22 | Patient_08 | IE | M | 19 |
| S3 Fig Panel A | 11 | IE, N=22 | Patient_09 | IE | M | 19 |
| S3 Fig Panel A | 12 | IE, N=22 | Patient_10 | IE | M | 20 |
| S3 Fig Panel A | 13 | IE, N=22 | Patient_11 | IE | M | 20 |
| S3 Fig Panel A | 14 | IE, N=22 | Patient_12 | IE | M | 21 |
| S3 Fig Panel A | 15 | IE, N=22 | Patient_13 | IE | M | 22 |
| S3 Fig Panel A | 16 | IE, N=22 | Patient_14 | IE | M | 24 |
| S3 Fig Panel A | 17 | IE, N=22 | Patient_15 | IE | M | 24 |
| S3 Fig Panel A | 18 | IE, N=22 | Patient_16 | IE | M | 27 |
| S3 Fig Panel A | 19 | IE, N=22 | Patient_17 | IE | M | 31 |
| S3 Fig Panel A | 20 | IE, N=22 | Patient_18 | IE | M | 33 |
| S3 Fig Panel A | 21 | IE, N=22 | Patient_19 | IE | M | 33 |
| S3 Fig Panel A | 22 | IE, N=22 | Patient_20 | IE | M | 40 |
| S3 Fig Panel A | 23 | IE, N=22 | Patient_21 | IE | M | 46 |
| S3 Fig Panel A | 24 | SCG, N=22 | Patient_34 | SCG | F | 18 |
| S3 Fig Panel A | 25 | SCG, N=22 | Patient_35 | SCG | F | 29 |
| S3 Fig Panel A | 26 | SCG, N=22 | Patient_36 | SCG | F | 31 |
| S3 Fig Panel A | 27 | SCG, N=22 | Patient_37 | SCG | F | 48 |
| S3 Fig Panel A | 28 | SCG, N=22 | Patient_38 | SCG | M | 18 |
| S3 Fig Panel A | 29 | SCG, N=22 | Patient_39 | SCG | M | 18 |
| S3 Fig Panel A | 30 | SCG, N=22 | Patient_40 | SCG | M | 19 |
| S3 Fig Panel A | 31 | SCG, N=22 | Patient_41 | SCG | M | 20 |
| S3 Fig Panel A | 32 | SCG, N=22 | Patient_42 | SCG | M | 23 |
| S3 Fig Panel A | 33 | SCG, N=22 | Patient_43 | SCG | M | 26 |
| S3 Fig Panel A | 34 | SCG, N=22 | Patient_44 | SCG | M | 26 |
| S3 Fig Panel A | 35 | SCG, N=22 | Patient_45 | SCG | M | 28 |
| S3 Fig Panel A | 36 | SCG, N=22 | Patient_46 | SCG | M | 29 |
| S3 Fig Panel A | 37 | SCG, N=22 | Patient_33 | SCG | M | 30 |
| S3 Fig Panel A | 38 | SCG, N=22 | Patient_47 | SCG | M | 33 |
| S3 Fig Panel A | 39 | SCG, N=22 | Patient_48 | SCG | M | 35 |
| S3 Fig Panel A | 40 | SCG, N=22 | Patient_49 | SCG | M | 39 |
| S3 Fig Panel A | 41 | SCG, N=22 | Patient_32 | SCG | M | 42 |
| S3 Fig Panel A | 42 | SCG, N=22 | Patient_50 | SCG | M | 42 |
| S3 Fig Panel A | 43 | SCG, N=22 | Patient_51 | SCG | M | 42 |
| S3 Fig Panel A | 44 | SCG, N=22 | Patient_52 | SCG | M | 45 |
| S3 Fig Panel A | 45 | SCG, N=22 | Patient_53 | SCG | M | 48 |
| S3 Fig Panel A | 46 | SCG, N=22 | Patient_54 | SCG | M | 48 |
| S3 Fig Panel B | 47 | RND IE, N=23 | Patient_01 | IE | F | 19 |
| S3 Fig Panel B | 48 | RND IE, N=23 | Patient_03 | IE | F | 21 |
| S3 Fig Panel B | 49 | RND IE, N=23 | Patient_04 | IE | F | 23 |
| S3 Fig Panel B | 50 | RND IE, N=23 | Patient_35 | SCG | F | 29 |
| S3 Fig Panel B | 51 | RND IE, N=23 | Patient_06 | IE | F | 30 |
| S3 Fig Panel B | 52 | RND IE, N=23 | Patient_28 | IE | F | 40 |
| S3 Fig Panel B | 53 | RND IE, N=23 | Patient_37 | SCG | F | 48 |
| S3 Fig Panel B | 54 | RND IE, N=23 | Patient_39 | SCG | M | 18 |
| S3 Fig Panel B | 55 | RND IE, N=23 | Patient_09 | IE | M | 19 |
| S3 Fig Panel B | 56 | RND IE, N=23 | Patient_11 | IE | M | 20 |
| S3 Fig Panel B | 57 | RND IE, N=23 | Patient_41 | SCG | M | 20 |
| S3 Fig Panel B | 58 | RND IE, N=23 | Patient_13 | IE | M | 22 |
| S3 Fig Panel B | 59 | RND IE, N=23 | Patient_15 | IE | M | 24 |
| S3 Fig Panel B | 60 | RND IE, N=23 | Patient_43 | SCG | M | 26 |
| S3 Fig Panel B | 61 | RND IE, N=23 | Patient_45 | SCG | M | 28 |
| S3 Fig Panel B | 62 | RND IE, N=23 | Patient_33 | SCG | M | 30 |
| S3 Fig Panel B | 63 | RND IE, N=23 | Patient_17 | IE | M | 31 |
| S3 Fig Panel B | 64 | RND IE, N=23 | Patient_19 | IE | M | 33 |
| S3 Fig Panel B | 65 | RND IE, N=23 | Patient_48 | SCG | M | 35 |
| S3 Fig Panel B | 66 | RND IE, N=23 | Patient_32 | SCG | M | 42 |
| S3 Fig Panel B | 67 | RND IE, N=23 | Patient_51 | SCG | M | 42 |
| S3 Fig Panel B | 68 | RND IE, N=23 | Patient_21 | IE | M | 46 |
| S3 Fig Panel B | 69 | RND IE, N=23 | Patient_53 | SCG | M | 48 |
| S3 Fig Panel B | 70 | RND SCG, N=23 | Patient_34 | SCG | F | 18 |
| S3 Fig Panel B | 71 | RND SCG, N=23 | Patient_02 | IE | F | 19 |
| S3 Fig Panel B | 72 | RND SCG, N=23 | Patient_29 | IE | F | 22 |
| S3 Fig Panel B | 73 | RND SCG, N=23 | Patient_05 | IE | F | 28 |
| S3 Fig Panel B | 74 | RND SCG, N=23 | Patient_36 | SCG | F | 31 |
| S3 Fig Panel B | 75 | RND SCG, N=23 | Patient_07 | IE | F | 32 |
| S3 Fig Panel B | 76 | RND SCG, N=23 | Patient_38 | SCG | M | 18 |
| S3 Fig Panel B | 77 | RND SCG, N=23 | Patient_08 | IE | M | 19 |
| S3 Fig Panel B | 78 | RND SCG, N=23 | Patient_40 | SCG | M | 19 |
| S3 Fig Panel B | 79 | RND SCG, N=23 | Patient_10 | IE | M | 20 |
| S3 Fig Panel B | 80 | RND SCG, N=23 | Patient_12 | IE | M | 21 |
| S3 Fig Panel B | 81 | RND SCG, N=23 | Patient_42 | SCG | M | 23 |
| S3 Fig Panel B | 82 | RND SCG, N=23 | Patient_14 | IE | M | 24 |
| S3 Fig Panel B | 83 | RND SCG, N=23 | Patient_44 | SCG | M | 26 |
| S3 Fig Panel B | 84 | RND SCG, N=23 | Patient_16 | IE | M | 27 |
| S3 Fig Panel B | 85 | RND SCG, N=23 | Patient_46 | SCG | M | 29 |
| S3 Fig Panel B | 86 | RND SCG, N=23 | Patient_18 | IE | M | 33 |
| S3 Fig Panel B | 87 | RND SCG, N=23 | Patient_47 | SCG | M | 33 |
| S3 Fig Panel B | 88 | RND SCG, N=23 | Patient_49 | SCG | M | 39 |
| S3 Fig Panel B | 89 | RND SCG, N=23 | Patient_20 | IE | M | 40 |
| S3 Fig Panel B | 90 | RND SCG, N=23 | Patient_50 | SCG | M | 42 |
| S3 Fig Panel B | 91 | RND SCG, N=23 | Patient_52 | SCG | M | 45 |
| S3 Fig Panel B | 92 | RND SCG, N=23 | Patient_54 | SCG | M | 48 |
| S3 Fig Panel c | 1 | blind SCG, N=6 | Patient_31 | SCG | F | 20 |
| S3 Fig Panel c | 2 | blind SCG, N=6 | Patient_30 | SCG | M | 21 |
| S3 Fig Panel c | 3 | blind SCG, N=6 | Patient_58 | SCG | M | 40 |
| S3 Fig Panel c | 4 | blind SCG, N=6 | Patient_56 | SCG | M | 23 |
| S3 Fig Panel c | 5 | blind SCG, N=6 | Patient_55 | SCG | M | 18 |
| S3 Fig Panel c | 6 | blind SCG, N=6 | Patient_57 | SCG | M | 24 |
| S3 Fig Panel c | 7 | blind IE, N=6 | Patient_23 | IE | M | 22 |
| S3 Fig Panel c | 8 | blind IE, N=6 | Patient_25 | IE | M | 30 |
| S3 Fig Panel c | 9 | blind IE, N=6 | Patient_24 | IE | M | 23 |
| S3 Fig Panel c | 10 | blind IE, N=6 | Patient_22 | IE | F | 34 |
| S3 Fig Panel c | 11 | blind IE, N=6 | Patient_27 | IE | M | 26 |
| S3 Fig Panel c | 12 | blind IE, N=6 | Patient_26 | IE | M | 33 |
| Table 3, Fig 4, Fig 5, S2 Fig, S3 Fig, S1 Table | - | MS/MS IE, N=10 | Patient_12 | IE | M | 21 |
| Table 3, Fig 4, Fig 5, S2 Fig, S3 Fig, S1 Table | - | MS/MS IE, N=10 | Patient_28 | IE | F | 40 |
| Table 3, Fig 4, Fig 5, S2 Fig, S3 Fig, S1 Table | - | MS/MS IE, N=10 | Patient_01 | IE | F | 19 |
| Table 3, Fig 4, Fig 5, S2 Fig, S3 Fig, S1 Table | - | MS/MS IE, N=10 | Patient_25 | IE | M | 30 |
| Table 3, Fig 4, Fig 5, S2 Fig, S3 Fig, S1 Table | - | MS/MS IE, N=10 | Patient_06 | IE | F | 30 |
| Table 3, Fig 4, Fig 5, S2 Fig, S3 Fig, S1 Table | - | MS/MS IE, N=10 | Patient_08 | IE | M | 19 |
| Table 3, Fig 4, Fig 5, S2 Fig, S3 Fig, S1 Table | - | MS/MS IE, N=10 | Patient_05 | IE | F | 28 |
| Table 3, Fig 4, Fig 5, S2 Fig, S3 Fig, S1 Table | - | MS/MS IE, N=10 | Patient_02 | IE | F | 19 |
| Table 3, Fig 4, Fig 5, S2 Fig, S3 Fig, S1 Table | - | MS/MS IE, N=10 | Patient_17 | IE | M | 31 |
| Table 3, Fig 4, Fig 5, S2 Fig, S3 Fig, S1 Table | - | MS/MS IE, N=10 | Patient_03 | IE | F | 21 |
| Table 3, Fig 4, Fig 5, S2 Fig, S3 Fig, S1 Table | - | MS/MS SCG, N=10 | Patient_46 | SCG | M | 29 |
| Table 3, Fig 4, Fig 5, S2 Fig, S3 Fig, S1 Table | - | MS/MS SCG, N=10 | Patient_31 | SCG | F | 20 |
| Table 3, Fig 4, Fig 5, S2 Fig, S3 Fig, S1 Table | - | MS/MS SCG, N=10 | Patient_35 | SCG | F | 29 |
| Table 3, Fig 4, Fig 5, S2 Fig, S3 Fig, S1 Table | - | MS/MS SCG, N=10 | Patient_33 | SCG | M | 30 |
| Table 3, Fig 4, Fig 5, S2 Fig, S3 Fig, S1 Table | - | MS/MS SCG, N=10 | Patient_37 | SCG | F | 48 |
| Table 3, Fig 4, Fig 5, S2 Fig, S3 Fig, S1 Table | - | MS/MS SCG, N=10 | Patient_36 | SCG | F | 31 |
| Table 3, Fig 4, Fig 5, S2 Fig, S3 Fig, S1 Table | - | MS/MS SCG, N=10 | Patient_41 | SCG | M | 20 |
| Table 3, Fig 4, Fig 5, S2 Fig, S3 Fig, S1 Table | - | MS/MS SCG, N=10 | Patient_34 | SCG | F | 18 |
| Table 3, Fig 4, Fig 5, S2 Fig, S3 Fig, S1 Table | - | MS/MS SCG, N=10 | Patient_54 | SCG | M | 48 |
| Table 3, Fig 4, Fig 5, S2 Fig, S3 Fig, S1 Table | - | MS/MS SCG, N=10 | Patient_39 | SCG | M | 18 |
