# Supplementary material for: Distinguishing patients with idiopathic epilepsy from solitary cysticercus granuloma epilepsy and biochemical phenotype assessment using a serum biomolecule profiling platform
Source: PLoS One. 2020 Aug 21;15(8):e0237064. doi: 10.1371/journal.pone.0237064 (PMC7527271; doi:10.1371/journal.pone.0237064)
Supplement: S3 Table — (DOCX) [file pone.0237064.s006.docx]

**S4 Table. Peaks utilized for each Figure: ESI-Ion-Trap MS and single quad-desktop MS.**

| **m/Z peaks used for classification** | **Groups tested** | **Figure and panel** | **Mass Spectrometer** |
| --- | --- | --- | --- |
| 501 | SCG, N=29 vs IE, N=29 | Fig 1 panel B, Figure 2 panel a, Figure 2 panel b | LCQ ADVANTAGE |
| 505 | SCG, N=29 vs IE, N=29 | Fig 1 panel B, Figure 2 panel a, Figure 2 panel b | LCQ ADVANTAGE |
| 558 | SCG, N=29 vs IE, N=29 | Fig 1 panel B, Figure 2 panel a, Figure 2 panel b | LCQ ADVANTAGE |
| 575 | SCG, N=29 vs IE, N=29 | Fig 1 panel B, Figure 2 panel a, Figure 2 panel b | LCQ ADVANTAGE |
| 578 | SCG, N=29 vs IE, N=29 | Fig 1 panel B, Figure 2 panel a, Figure 2 panel b | LCQ ADVANTAGE |
| 585 | SCG, N=29 vs IE, N=29 | Fig 1 panel B, Figure 2 panel a, Figure 2 panel b | LCQ ADVANTAGE |
| 588 | SCG, N=29 vs IE, N=29 | Fig 1 panel B, Figure 2 panel a, Figure 2 panel b | LCQ ADVANTAGE |
| 589 | SCG, N=29 vs IE, N=29 | Fig 1 panel B, Figure 2 panel a, Figure 2 panel b | LCQ ADVANTAGE |
| 606 | SCG, N=29 vs IE, N=29 | Fig 1 panel B, Figure 2 panel a, Figure 2 panel b | LCQ ADVANTAGE |
| 609 | SCG, N=29 vs IE, N=29 | Fig 1 panel B, Figure 2 panel a, Figure 2 panel b | LCQ ADVANTAGE |
| 619 | SCG, N=29 vs IE, N=29 | Fig 1 panel B, Figure 2 panel a, Figure 2 panel b | LCQ ADVANTAGE |
| 652 | SCG, N=29 vs IE, N=29 | Fig 1 panel B, Figure 2 panel a, Figure 2 panel b | LCQ ADVANTAGE |
| 688 | SCG, N=29 vs IE, N=29 | Fig 1 panel B, Figure 2 panel a, Figure 2 panel b | LCQ ADVANTAGE |
| 716 | SCG, N=29 vs IE, N=29 | Fig 1 panel B, Figure 2 panel a, Figure 2 panel b | LCQ ADVANTAGE |
| 720 | SCG, N=29 vs IE, N=29 | Fig 1 panel B, Figure 2 panel a, Figure 2 panel b | LCQ ADVANTAGE |
| 722 | SCG, N=29 vs IE, N=29 | Fig 1 panel B, Figure 2 panel a, Figure 2 panel b | LCQ ADVANTAGE |
| 746 | SCG, N=29 vs IE, N=29 | Fig 1 panel B, Figure 2 panel a, Figure 2 panel b | LCQ ADVANTAGE |
| 749 | SCG, N=29 vs IE, N=29 | Fig 1 panel B, Figure 2 panel a, Figure 2 panel b | LCQ ADVANTAGE |
| 764 | SCG, N=29 vs IE, N=29 | Fig 1 panel B, Figure 2 panel a, Figure 2 panel b | LCQ ADVANTAGE |
| 781 | SCG, N=29 vs IE, N=29 | Fig 1 panel B, Figure 2 panel a, Figure 2 panel b | LCQ ADVANTAGE |
| 791 | SCG, N=29 vs IE, N=29 | Fig 1 panel B, Figure 2 panel a, Figure 2 panel b | LCQ ADVANTAGE |
| 811 | SCG, N=29 vs IE, N=29 | Fig 1 panel B, Figure 2 panel a, Figure 2 panel b | LCQ ADVANTAGE |
| 813 | SCG, N=29 vs IE, N=29 | Fig 1 panel B, Figure 2 panel a, Figure 2 panel b | LCQ ADVANTAGE |
| 820 | SCG, N=29 vs IE, N=29 | Fig 1 panel B, Figure 2 panel a, Figure 2 panel b | LCQ ADVANTAGE |
| 824 | SCG, N=29 vs IE, N=29 | Fig 1 panel B, Figure 2 panel a, Figure 2 panel b | LCQ ADVANTAGE |
| 852 | SCG, N=29 vs IE, N=29 | Fig 1 panel B, Figure 2 panel a, Figure 2 panel b | LCQ ADVANTAGE |
| 856 | SCG, N=29 vs IE, N=29 | Fig 1 panel B, Figure 2 panel a, Figure 2 panel b | LCQ ADVANTAGE |
| 865 | SCG, N=29 vs IE, N=29 | Fig 1 panel B, Figure 2 panel a, Figure 2 panel b | LCQ ADVANTAGE |
| 867 | SCG, N=29 vs IE, N=29 | Fig 1 panel B, Figure 2 panel a, Figure 2 panel b | LCQ ADVANTAGE |
| 894 | SCG, N=29 vs IE, N=29 | Fig 1 panel B, Figure 2 panel a, Figure 2 panel b | LCQ ADVANTAGE |
| 899 | SCG, N=29 vs IE, N=29 | Fig 1 panel B, Figure 2 panel a, Figure 2 panel b | LCQ ADVANTAGE |
| 914 | SCG, N=29 vs IE, N=29 | Fig 1 panel B, Figure 2 panel a, Figure 2 panel b | LCQ ADVANTAGE |
| 916 | SCG, N=29 vs IE, N=29 | Fig 1 panel B, Figure 2 panel a, Figure 2 panel b | LCQ ADVANTAGE |
| 941 | SCG, N=29 vs IE, N=29 | Fig 1 panel B, Figure 2 panel a, Figure 2 panel b | LCQ ADVANTAGE |
| 945 | SCG, N=29 vs IE, N=29 | Fig 1 panel B, Figure 2 panel a, Figure 2 panel b | LCQ ADVANTAGE |
| 946 | SCG, N=29 vs IE, N=29 | Fig 1 panel B, Figure 2 panel a, Figure 2 panel b | LCQ ADVANTAGE |
| 963 | SCG, N=29 vs IE, N=29 | Fig 1 panel B, Figure 2 panel a, Figure 2 panel b | LCQ ADVANTAGE |
| 964 | SCG, N=29 vs IE, N=29 | Fig 1 panel B, Figure 2 panel a, Figure 2 panel b | LCQ ADVANTAGE |
| 965 | SCG, N=29 vs IE, N=29 | Fig 1 panel B, Figure 2 panel a, Figure 2 panel b | LCQ ADVANTAGE |
| 979 | SCG, N=29 vs IE, N=29 | Fig 1 panel B, Figure 2 panel a, Figure 2 panel b | LCQ ADVANTAGE |
| 999 | SCG, N=29 vs IE, N=29 | Fig 1 panel B, Figure 2 panel a, Figure 2 panel b | LCQ ADVANTAGE |
| 1000 | SCG, N=29 vs IE, N=29 | Fig 1 panel B, Figure 2 panel a, Figure 2 panel b | LCQ ADVANTAGE |
| 1014 | SCG, N=29 vs IE, N=29 | Fig 1 panel B, Figure 2 panel a, Figure 2 panel b | LCQ ADVANTAGE |
| 1016 | SCG, N=29 vs IE, N=29 | Fig 1 panel B, Figure 2 panel a, Figure 2 panel b | LCQ ADVANTAGE |
| 1040 | SCG, N=29 vs IE, N=29 | Fig 1 panel B, Figure 2 panel a, Figure 2 panel b | LCQ ADVANTAGE |
| 1045 | SCG, N=29 vs IE, N=29 | Fig 1 panel B, Figure 2 panel a, Figure 2 panel b | LCQ ADVANTAGE |
| 1047 | SCG, N=29 vs IE, N=29 | Fig 1 panel B, Figure 2 panel a, Figure 2 panel b | LCQ ADVANTAGE |
| 1049 | SCG, N=29 vs IE, N=29 | Fig 1 panel B, Figure 2 panel a, Figure 2 panel b | LCQ ADVANTAGE |
| 1050 | SCG, N=29 vs IE, N=29 | Fig 1 panel B, Figure 2 panel a, Figure 2 panel b | LCQ ADVANTAGE |
| 1053 | SCG, N=29 vs IE, N=29 | Fig 1 panel B, Figure 2 panel a, Figure 2 panel b | LCQ ADVANTAGE |
| 1064 | SCG, N=29 vs IE, N=29 | Fig 1 panel B, Figure 2 panel a, Figure 2 panel b | LCQ ADVANTAGE |
| 1067 | SCG, N=29 vs IE, N=29 | Fig 1 panel B, Figure 2 panel a, Figure 2 panel b | LCQ ADVANTAGE |
| 1069 | SCG, N=29 vs IE, N=29 | Fig 1 panel B, Figure 2 panel a, Figure 2 panel b | LCQ ADVANTAGE |
| 1083 | SCG, N=29 vs IE, N=29 | Fig 1 panel B, Figure 2 panel a, Figure 2 panel b | LCQ ADVANTAGE |
| 1106 | SCG, N=29 vs IE, N=29 | Fig 1 panel B, Figure 2 panel a, Figure 2 panel b | LCQ ADVANTAGE |
| 1122 | SCG, N=29 vs IE, N=29 | Fig 1 panel B, Figure 2 panel a, Figure 2 panel b | LCQ ADVANTAGE |
| 1154 | SCG, N=29 vs IE, N=29 | Fig 1 panel B, Figure 2 panel a, Figure 2 panel b | LCQ ADVANTAGE |
| 1173 | SCG, N=29 vs IE, N=29 | Fig 1 panel B, Figure 2 panel a, Figure 2 panel b | LCQ ADVANTAGE |
| 1401 | SCG, N=29 vs IE, N=29 | Fig 1 panel B, Figure 2 panel a, Figure 2 panel b | LCQ ADVANTAGE |
| 1403 | SCG, N=29 vs IE, N=29 | Fig 1 panel B, Figure 2 panel a, Figure 2 panel b | LCQ ADVANTAGE |
| 1405 | SCG, N=29 vs IE, N=29 | Fig 1 panel B, Figure 2 panel a, Figure 2 panel b | LCQ ADVANTAGE |
| 1408 | SCG, N=29 vs IE, N=29 | Fig 1 panel B, Figure 2 panel a, Figure 2 panel b | LCQ ADVANTAGE |
| 1439 | SCG, N=29 vs IE, N=29 | Fig 1 panel B, Figure 2 panel a, Figure 2 panel b | LCQ ADVANTAGE |
| 1463 | SCG, N=29 vs IE, N=29 | Fig 1 panel B, Figure 2 panel a, Figure 2 panel b | LCQ ADVANTAGE |
| 1472 | SCG, N=29 vs IE, N=29 | Fig 1 panel B, Figure 2 panel a, Figure 2 panel b | LCQ ADVANTAGE |
| 1489 | SCG, N=29 vs IE, N=29 | Fig 1 panel B, Figure 2 panel a, Figure 2 panel b | LCQ ADVANTAGE |
| 1494 | SCG, N=29 vs IE, N=29 | Fig 1 panel B, Figure 2 panel a, Figure 2 panel b | LCQ ADVANTAGE |
| 1504 | SCG, N=29 vs IE, N=29 | Fig 1 panel B, Figure 2 panel a, Figure 2 panel b | LCQ ADVANTAGE |
| 1513 | SCG, N=29 vs IE, N=29 | Fig 1 panel B, Figure 2 panel a, Figure 2 panel b | LCQ ADVANTAGE |
| 1523 | SCG, N=29 vs IE, N=29 | Fig 1 panel B, Figure 2 panel a, Figure 2 panel b | LCQ ADVANTAGE |
| 1528 | SCG, N=29 vs IE, N=29 | Fig 1 panel B, Figure 2 panel a, Figure 2 panel b | LCQ ADVANTAGE |
| 1553 | SCG, N=29 vs IE, N=29 | Fig 1 panel B, Figure 2 panel a, Figure 2 panel b | LCQ ADVANTAGE |
| 1566 | SCG, N=29 vs IE, N=29 | Fig 1 panel B, Figure 2 panel a, Figure 2 panel b | LCQ ADVANTAGE |
| 1568 | SCG, N=29 vs IE, N=29 | Fig 1 panel B, Figure 2 panel a, Figure 2 panel b | LCQ ADVANTAGE |
| 1569 | SCG, N=29 vs IE, N=29 | Fig 1 panel B, Figure 2 panel a, Figure 2 panel b | LCQ ADVANTAGE |
| 1580 | SCG, N=29 vs IE, N=29 | Fig 1 panel B, Figure 2 panel a, Figure 2 panel b | LCQ ADVANTAGE |
| 1584 | SCG, N=29 vs IE, N=29 | Fig 1 panel B, Figure 2 panel a, Figure 2 panel b | LCQ ADVANTAGE |
| 1586 | SCG, N=29 vs IE, N=29 | Fig 1 panel B, Figure 2 panel a, Figure 2 panel b | LCQ ADVANTAGE |
| 1587 | SCG, N=29 vs IE, N=29 | Fig 1 panel B, Figure 2 panel a, Figure 2 panel b | LCQ ADVANTAGE |
| 1589 | SCG, N=29 vs IE, N=29 | Fig 1 panel B, Figure 2 panel a, Figure 2 panel b | LCQ ADVANTAGE |
| 1592 | SCG, N=29 vs IE, N=29 | Fig 1 panel B, Figure 2 panel a, Figure 2 panel b | LCQ ADVANTAGE |
| 1594 | SCG, N=29 vs IE, N=29 | Fig 1 panel B, Figure 2 panel a, Figure 2 panel b | LCQ ADVANTAGE |
| 1605 | SCG, N=29 vs IE, N=29 | Fig 1 panel B, Figure 2 panel a, Figure 2 panel b | LCQ ADVANTAGE |
| 1609 | SCG, N=29 vs IE, N=29 | Fig 1 panel B, Figure 2 panel a, Figure 2 panel b | LCQ ADVANTAGE |
| 1619 | SCG, N=29 vs IE, N=29 | Fig 1 panel B, Figure 2 panel a, Figure 2 panel b | LCQ ADVANTAGE |
| 1656 | SCG, N=29 vs IE, N=29 | Fig 1 panel B, Figure 2 panel a, Figure 2 panel b | LCQ ADVANTAGE |
| 1666 | SCG, N=29 vs IE, N=29 | Fig 1 panel B, Figure 2 panel a, Figure 2 panel b | LCQ ADVANTAGE |
| 1672 | SCG, N=29 vs IE, N=29 | Fig 1 panel B, Figure 2 panel a, Figure 2 panel b | LCQ ADVANTAGE |
| 1681 | SCG, N=29 vs IE, N=29 | Fig 1 panel B, Figure 2 panel a, Figure 2 panel b | LCQ ADVANTAGE |
| 1682 | SCG, N=29 vs IE, N=29 | Fig 1 panel B, Figure 2 panel a, Figure 2 panel b | LCQ ADVANTAGE |
| 1689 | SCG, N=29 vs IE, N=29 | Fig 1 panel B, Figure 2 panel a, Figure 2 panel b | LCQ ADVANTAGE |
| 1691 | SCG, N=29 vs IE, N=29 | Fig 1 panel B, Figure 2 panel a, Figure 2 panel b | LCQ ADVANTAGE |
| 1704 | SCG, N=29 vs IE, N=29 | Fig 1 panel B, Figure 2 panel a, Figure 2 panel b | LCQ ADVANTAGE |
| 1705 | SCG, N=29 vs IE, N=29 | Fig 1 panel B, Figure 2 panel a, Figure 2 panel b | LCQ ADVANTAGE |
| 1713 | SCG, N=29 vs IE, N=29 | Fig 1 panel B, Figure 2 panel a, Figure 2 panel b | LCQ ADVANTAGE |
| 1735 | SCG, N=29 vs IE, N=29 | Fig 1 panel B, Figure 2 panel a, Figure 2 panel b | LCQ ADVANTAGE |
| 1737 | SCG, N=29 vs IE, N=29 | Fig 1 panel B, Figure 2 panel a, Figure 2 panel b | LCQ ADVANTAGE |
| 1760 | SCG, N=29 vs IE, N=29 | Fig 1 panel B, Figure 2 panel a, Figure 2 panel b | LCQ ADVANTAGE |
| 1762 | SCG, N=29 vs IE, N=29 | Fig 1 panel B, Figure 2 panel a, Figure 2 panel b | LCQ ADVANTAGE |
| 1763 | SCG, N=29 vs IE, N=29 | Fig 1 panel B, Figure 2 panel a, Figure 2 panel b | LCQ ADVANTAGE |
| 1772 | SCG, N=29 vs IE, N=29 | Fig 1 panel B, Figure 2 panel a, Figure 2 panel b | LCQ ADVANTAGE |
| 1795 | SCG, N=29 vs IE, N=29 | Fig 1 panel B, Figure 2 panel a, Figure 2 panel b | LCQ ADVANTAGE |
| 1797 | SCG, N=29 vs IE, N=29 | Fig 1 panel B, Figure 2 panel a, Figure 2 panel b | LCQ ADVANTAGE |
| 1813 | SCG, N=29 vs IE, N=29 | Fig 1 panel B, Figure 2 panel a, Figure 2 panel b | LCQ ADVANTAGE |
| 1825 | SCG, N=29 vs IE, N=29 | Fig 1 panel B, Figure 2 panel a, Figure 2 panel b | LCQ ADVANTAGE |
| 1838 | SCG, N=29 vs IE, N=29 | Fig 1 panel B, Figure 2 panel a, Figure 2 panel b | LCQ ADVANTAGE |
| 1842 | SCG, N=29 vs IE, N=29 | Fig 1 panel B, Figure 2 panel a, Figure 2 panel b | LCQ ADVANTAGE |
| 1843 | SCG, N=29 vs IE, N=29 | Fig 1 panel B, Figure 2 panel a, Figure 2 panel b | LCQ ADVANTAGE |
| 1847 | SCG, N=29 vs IE, N=29 | Fig 1 panel B, Figure 2 panel a, Figure 2 panel b | LCQ ADVANTAGE |
| 505 | LCQ Training sets SCG, N=23 vs IE, N=23 and Blind samples SCG, N=6 vs IE, N=6 | Fig 3 | LCQ ADVANTAGE |
| 558 | LCQ Training sets SCG, N=23 vs IE, N=23 and Blind samples SCG, N=6 vs IE, N=6 | Fig 3 | LCQ ADVANTAGE |
| 560 | LCQ Training sets SCG, N=23 vs IE, N=23 and Blind samples SCG, N=6 vs IE, N=6 | Fig 3 | LCQ ADVANTAGE |
| 569 | LCQ Training sets SCG, N=23 vs IE, N=23 and Blind samples SCG, N=6 vs IE, N=6 | Fig 3 | LCQ ADVANTAGE |
| 573 | LCQ Training sets SCG, N=23 vs IE, N=23 and Blind samples SCG, N=6 vs IE, N=6 | Fig 3 | LCQ ADVANTAGE |
| 575 | LCQ Training sets SCG, N=23 vs IE, N=23 and Blind samples SCG, N=6 vs IE, N=6 | Fig 3 | LCQ ADVANTAGE |
| 578 | LCQ Training sets SCG, N=23 vs IE, N=23 and Blind samples SCG, N=6 vs IE, N=6 | Fig 3 | LCQ ADVANTAGE |
| 585 | LCQ Training sets SCG, N=23 vs IE, N=23 and Blind samples SCG, N=6 vs IE, N=6 | Fig 3 | LCQ ADVANTAGE |
| 587 | LCQ Training sets SCG, N=23 vs IE, N=23 and Blind samples SCG, N=6 vs IE, N=6 | Fig 3 | LCQ ADVANTAGE |
| 588 | LCQ Training sets SCG, N=23 vs IE, N=23 and Blind samples SCG, N=6 vs IE, N=6 | Fig 3 | LCQ ADVANTAGE |
| 589 | LCQ Training sets SCG, N=23 vs IE, N=23 and Blind samples SCG, N=6 vs IE, N=6 | Fig 3 | LCQ ADVANTAGE |
| 600 | LCQ Training sets SCG, N=23 vs IE, N=23 and Blind samples SCG, N=6 vs IE, N=6 | Fig 3 | LCQ ADVANTAGE |
| 603 | LCQ Training sets SCG, N=23 vs IE, N=23 and Blind samples SCG, N=6 vs IE, N=6 | Fig 3 | LCQ ADVANTAGE |
| 617 | LCQ Training sets SCG, N=23 vs IE, N=23 and Blind samples SCG, N=6 vs IE, N=6 | Fig 3 | LCQ ADVANTAGE |
| 619 | LCQ Training sets SCG, N=23 vs IE, N=23 and Blind samples SCG, N=6 vs IE, N=6 | Fig 3 | LCQ ADVANTAGE |
| 652 | LCQ Training sets SCG, N=23 vs IE, N=23 and Blind samples SCG, N=6 vs IE, N=6 | Fig 3 | LCQ ADVANTAGE |
| 655 | LCQ Training sets SCG, N=23 vs IE, N=23 and Blind samples SCG, N=6 vs IE, N=6 | Fig 3 | LCQ ADVANTAGE |
| 678 | LCQ Training sets SCG, N=23 vs IE, N=23 and Blind samples SCG, N=6 vs IE, N=6 | Fig 3 | LCQ ADVANTAGE |
| 686 | LCQ Training sets SCG, N=23 vs IE, N=23 and Blind samples SCG, N=6 vs IE, N=6 | Fig 3 | LCQ ADVANTAGE |
| 688 | LCQ Training sets SCG, N=23 vs IE, N=23 and Blind samples SCG, N=6 vs IE, N=6 | Fig 3 | LCQ ADVANTAGE |
| 716 | LCQ Training sets SCG, N=23 vs IE, N=23 and Blind samples SCG, N=6 vs IE, N=6 | Fig 3 | LCQ ADVANTAGE |
| 820 | LCQ Training sets SCG, N=23 vs IE, N=23 and Blind samples SCG, N=6 vs IE, N=6 | Fig 3 | LCQ ADVANTAGE |
| 824 | LCQ Training sets SCG, N=23 vs IE, N=23 and Blind samples SCG, N=6 vs IE, N=6 | Fig 3 | LCQ ADVANTAGE |
| 852 | LCQ Training sets SCG, N=23 vs IE, N=23 and Blind samples SCG, N=6 vs IE, N=6 | Fig 3 | LCQ ADVANTAGE |
| 856 | LCQ Training sets SCG, N=23 vs IE, N=23 and Blind samples SCG, N=6 vs IE, N=6 | Fig 3 | LCQ ADVANTAGE |
| 865 | LCQ Training sets SCG, N=23 vs IE, N=23 and Blind samples SCG, N=6 vs IE, N=6 | Fig 3 | LCQ ADVANTAGE |
| 875 | LCQ Training sets SCG, N=23 vs IE, N=23 and Blind samples SCG, N=6 vs IE, N=6 | Fig 3 | LCQ ADVANTAGE |
| 894 | LCQ Training sets SCG, N=23 vs IE, N=23 and Blind samples SCG, N=6 vs IE, N=6 | Fig 3 | LCQ ADVANTAGE |
| 899 | LCQ Training sets SCG, N=23 vs IE, N=23 and Blind samples SCG, N=6 vs IE, N=6 | Fig 3 | LCQ ADVANTAGE |
| 914 | LCQ Training sets SCG, N=23 vs IE, N=23 and Blind samples SCG, N=6 vs IE, N=6 | Fig 3 | LCQ ADVANTAGE |
| 919 | LCQ Training sets SCG, N=23 vs IE, N=23 and Blind samples SCG, N=6 vs IE, N=6 | Fig 3 | LCQ ADVANTAGE |
| 934 | LCQ Training sets SCG, N=23 vs IE, N=23 and Blind samples SCG, N=6 vs IE, N=6 | Fig 3 | LCQ ADVANTAGE |
| 940 | LCQ Training sets SCG, N=23 vs IE, N=23 and Blind samples SCG, N=6 vs IE, N=6 | Fig 3 | LCQ ADVANTAGE |
| 942 | LCQ Training sets SCG, N=23 vs IE, N=23 and Blind samples SCG, N=6 vs IE, N=6 | Fig 3 | LCQ ADVANTAGE |
| 1106 | LCQ Training sets SCG, N=23 vs IE, N=23 and Blind samples SCG, N=6 vs IE, N=6 | Fig 3 | LCQ ADVANTAGE |
| 1114 | LCQ Training sets SCG, N=23 vs IE, N=23 and Blind samples SCG, N=6 vs IE, N=6 | Fig 3 | LCQ ADVANTAGE |
| 1118 | LCQ Training sets SCG, N=23 vs IE, N=23 and Blind samples SCG, N=6 vs IE, N=6 | Fig 3 | LCQ ADVANTAGE |
| 1122 | LCQ Training sets SCG, N=23 vs IE, N=23 and Blind samples SCG, N=6 vs IE, N=6 | Fig 3 | LCQ ADVANTAGE |
| 1154 | LCQ Training sets SCG, N=23 vs IE, N=23 and Blind samples SCG, N=6 vs IE, N=6 | Fig 3 | LCQ ADVANTAGE |
| 1173 | LCQ Training sets SCG, N=23 vs IE, N=23 and Blind samples SCG, N=6 vs IE, N=6 | Fig 3 | LCQ ADVANTAGE |
| 1175 | LCQ Training sets SCG, N=23 vs IE, N=23 and Blind samples SCG, N=6 vs IE, N=6 | Fig 3 | LCQ ADVANTAGE |
| 1196 | LCQ Training sets SCG, N=23 vs IE, N=23 and Blind samples SCG, N=6 vs IE, N=6 | Fig 3 | LCQ ADVANTAGE |
| 1197 | LCQ Training sets SCG, N=23 vs IE, N=23 and Blind samples SCG, N=6 vs IE, N=6 | Fig 3 | LCQ ADVANTAGE |
| 1227 | LCQ Training sets SCG, N=23 vs IE, N=23 and Blind samples SCG, N=6 vs IE, N=6 | Fig 3 | LCQ ADVANTAGE |
| 1233 | LCQ Training sets SCG, N=23 vs IE, N=23 and Blind samples SCG, N=6 vs IE, N=6 | Fig 3 | LCQ ADVANTAGE |
| 1247 | LCQ Training sets SCG, N=23 vs IE, N=23 and Blind samples SCG, N=6 vs IE, N=6 | Fig 3 | LCQ ADVANTAGE |
| 1306 | LCQ Training sets SCG, N=23 vs IE, N=23 and Blind samples SCG, N=6 vs IE, N=6 | Fig 3 | LCQ ADVANTAGE |
| 1523 | LCQ Training sets SCG, N=23 vs IE, N=23 and Blind samples SCG, N=6 vs IE, N=6 | Fig 3 | LCQ ADVANTAGE |
| 1542 | LCQ Training sets SCG, N=23 vs IE, N=23 and Blind samples SCG, N=6 vs IE, N=6 | Fig 3 | LCQ ADVANTAGE |
| 1553 | LCQ Training sets SCG, N=23 vs IE, N=23 and Blind samples SCG, N=6 vs IE, N=6 | Fig 3 | LCQ ADVANTAGE |
| 1568 | LCQ Training sets SCG, N=23 vs IE, N=23 and Blind samples SCG, N=6 vs IE, N=6 | Fig 3 | LCQ ADVANTAGE |
| 1569 | LCQ Training sets SCG, N=23 vs IE, N=23 and Blind samples SCG, N=6 vs IE, N=6 | Fig 3 | LCQ ADVANTAGE |
| 1581 | LCQ Training sets SCG, N=23 vs IE, N=23 and Blind samples SCG, N=6 vs IE, N=6 | Fig 3 | LCQ ADVANTAGE |
| 1586 | LCQ Training sets SCG, N=23 vs IE, N=23 and Blind samples SCG, N=6 vs IE, N=6 | Fig 3 | LCQ ADVANTAGE |
| 1639 | LCQ Training sets SCG, N=23 vs IE, N=23 and Blind samples SCG, N=6 vs IE, N=6 | Fig 3 | LCQ ADVANTAGE |
| 1656 | LCQ Training sets SCG, N=23 vs IE, N=23 and Blind samples SCG, N=6 vs IE, N=6 | Fig 3 | LCQ ADVANTAGE |
| 1666 | LCQ Training sets SCG, N=23 vs IE, N=23 and Blind samples SCG, N=6 vs IE, N=6 | Fig 3 | LCQ ADVANTAGE |
| 1672 | LCQ Training sets SCG, N=23 vs IE, N=23 and Blind samples SCG, N=6 vs IE, N=6 | Fig 3 | LCQ ADVANTAGE |
| 1681 | LCQ Training sets SCG, N=23 vs IE, N=23 and Blind samples SCG, N=6 vs IE, N=6 | Fig 3 | LCQ ADVANTAGE |
| 1682 | LCQ Training sets SCG, N=23 vs IE, N=23 and Blind samples SCG, N=6 vs IE, N=6 | Fig 3 | LCQ ADVANTAGE |
| 1689 | LCQ Training sets SCG, N=23 vs IE, N=23 and Blind samples SCG, N=6 vs IE, N=6 | Fig 3 | LCQ ADVANTAGE |
| 1704 | LCQ Training sets SCG, N=23 vs IE, N=23 and Blind samples SCG, N=6 vs IE, N=6 | Fig 3 | LCQ ADVANTAGE |
| 1760 | LCQ Training sets SCG, N=23 vs IE, N=23 and Blind samples SCG, N=6 vs IE, N=6 | Fig 3 | LCQ ADVANTAGE |
| 1763 | LCQ Training sets SCG, N=23 vs IE, N=23 and Blind samples SCG, N=6 vs IE, N=6 | Fig 3 | LCQ ADVANTAGE |
| 1772 | LCQ Training sets SCG, N=23 vs IE, N=23 and Blind samples SCG, N=6 vs IE, N=6 | Fig 3 | LCQ ADVANTAGE |
| 1792 | LCQ Training sets SCG, N=23 vs IE, N=23 and Blind samples SCG, N=6 vs IE, N=6 | Fig 3 | LCQ ADVANTAGE |
| 1793 | LCQ Training sets SCG, N=23 vs IE, N=23 and Blind samples SCG, N=6 vs IE, N=6 | Fig 3 | LCQ ADVANTAGE |
| 1795 | LCQ Training sets SCG, N=23 vs IE, N=23 and Blind samples SCG, N=6 vs IE, N=6 | Fig 3 | LCQ ADVANTAGE |
| 1797 | LCQ Training sets SCG, N=23 vs IE, N=23 and Blind samples SCG, N=6 vs IE, N=6 | Fig 3 | LCQ ADVANTAGE |
| 1813 | LCQ Training sets SCG, N=23 vs IE, N=23 and Blind samples SCG, N=6 vs IE, N=6 | Fig 3 | LCQ ADVANTAGE |
| 1814 | LCQ Training sets SCG, N=23 vs IE, N=23 and Blind samples SCG, N=6 vs IE, N=6 | Fig 3 | LCQ ADVANTAGE |
| 1838 | LCQ Training sets SCG, N=23 vs IE, N=23 and Blind samples SCG, N=6 vs IE, N=6 | Fig 3 | LCQ ADVANTAGE |
| 1924 | LCQ Training sets SCG, N=23 vs IE, N=23 and Blind samples SCG, N=6 vs IE, N=6 | Fig 3 | LCQ ADVANTAGE |
| 1957 | LCQ Training sets SCG, N=23 vs IE, N=23 and Blind samples SCG, N=6 vs IE, N=6 | Fig 3 | LCQ ADVANTAGE |
| 1958 | LCQ Training sets SCG, N=23 vs IE, N=23 and Blind samples SCG, N=6 vs IE, N=6 | Fig 3 | LCQ ADVANTAGE |
| 1968 | LCQ Training sets SCG, N=23 vs IE, N=23 and Blind samples SCG, N=6 vs IE, N=6 | Fig 3 | LCQ ADVANTAGE |
| 1970 | LCQ Training sets SCG, N=23 vs IE, N=23 and Blind samples SCG, N=6 vs IE, N=6 | Fig 3 | LCQ ADVANTAGE |
| 1979 | LCQ Training sets SCG, N=23 vs IE, N=23 and Blind samples SCG, N=6 vs IE, N=6 | Fig 3 | LCQ ADVANTAGE |
| 1985 | LCQ Training sets SCG, N=23 vs IE, N=23 and Blind samples SCG, N=6 vs IE, N=6 | Fig 3 | LCQ ADVANTAGE |
| 513 | Advion SCG, N=29 vs IE, N=29 | S1 Fig Panel A and S1 Fig Panel B | Advion |
| 514 | Advion SCG, N=29 vs IE, N=29 | S1 Fig Panel A and S1 Fig Panel B | Advion |
| 546 | Advion SCG, N=29 vs IE, N=29 | S1 Fig Panel A and S1 Fig Panel B | Advion |
| 561 | Advion SCG, N=29 vs IE, N=29 | S1 Fig Panel A and S1 Fig Panel B | Advion |
| 567 | Advion SCG, N=29 vs IE, N=29 | S1 Fig Panel A and S1 Fig Panel B | Advion |
| 588 | Advion SCG, N=29 vs IE, N=29 | S1 Fig Panel A and S1 Fig Panel B | Advion |
| 607 | Advion SCG, N=29 vs IE, N=29 | S1 Fig Panel A and S1 Fig Panel B | Advion |
| 614 | Advion SCG, N=29 vs IE, N=29 | S1 Fig Panel A and S1 Fig Panel B | Advion |
| 618 | Advion SCG, N=29 vs IE, N=29 | S1 Fig Panel A and S1 Fig Panel B | Advion |
| 626 | Advion SCG, N=29 vs IE, N=29 | S1 Fig Panel A and S1 Fig Panel B | Advion |
| 664 | Advion SCG, N=29 vs IE, N=29 | S1 Fig Panel A and S1 Fig Panel B | Advion |
| 677 | Advion SCG, N=29 vs IE, N=29 | S1 Fig Panel A and S1 Fig Panel B | Advion |
| 695 | Advion SCG, N=29 vs IE, N=29 | S1 Fig Panel A and S1 Fig Panel B | Advion |
| 698 | Advion SCG, N=29 vs IE, N=29 | S1 Fig Panel A and S1 Fig Panel B | Advion |
| 699 | Advion SCG, N=29 vs IE, N=29 | S1 Fig Panel A and S1 Fig Panel B | Advion |
| 712 | Advion SCG, N=29 vs IE, N=29 | S1 Fig Panel A and S1 Fig Panel B | Advion |
| 731 | Advion SCG, N=29 vs IE, N=29 | S1 Fig Panel A and S1 Fig Panel B | Advion |
| 733 | Advion SCG, N=29 vs IE, N=29 | S1 Fig Panel A and S1 Fig Panel B | Advion |
| 753 | Advion SCG, N=29 vs IE, N=29 | S1 Fig Panel A and S1 Fig Panel B | Advion |
| 768 | Advion SCG, N=29 vs IE, N=29 | S1 Fig Panel A and S1 Fig Panel B | Advion |
| 771 | Advion SCG, N=29 vs IE, N=29 | S1 Fig Panel A and S1 Fig Panel B | Advion |
| 774 | Advion SCG, N=29 vs IE, N=29 | S1 Fig Panel A and S1 Fig Panel B | Advion |
| 789 | Advion SCG, N=29 vs IE, N=29 | S1 Fig Panel A and S1 Fig Panel B | Advion |
| 838 | Advion SCG, N=29 vs IE, N=29 | S1 Fig Panel A and S1 Fig Panel B | Advion |
| 864 | Advion SCG, N=29 vs IE, N=29 | S1 Fig Panel A and S1 Fig Panel B | Advion |
| 874 | Advion SCG, N=29 vs IE, N=29 | S1 Fig Panel A and S1 Fig Panel B | Advion |
| 888 | Advion SCG, N=29 vs IE, N=29 | S1 Fig Panel A and S1 Fig Panel B | Advion |
| 910 | Advion SCG, N=29 vs IE, N=29 | S1 Fig Panel A and S1 Fig Panel B | Advion |
| 917 | Advion SCG, N=29 vs IE, N=29 | S1 Fig Panel A and S1 Fig Panel B | Advion |
| 918 | Advion SCG, N=29 vs IE, N=29 | S1 Fig Panel A and S1 Fig Panel B | Advion |
| 926 | Advion SCG, N=29 vs IE, N=29 | S1 Fig Panel A and S1 Fig Panel B | Advion |
| 941 | Advion SCG, N=29 vs IE, N=29 | S1 Fig Panel A and S1 Fig Panel B | Advion |
| 951 | Advion SCG, N=29 vs IE, N=29 | S1 Fig Panel A and S1 Fig Panel B | Advion |
| 958 | Advion SCG, N=29 vs IE, N=29 | S1 Fig Panel A and S1 Fig Panel B | Advion |
| 976 | Advion SCG, N=29 vs IE, N=29 | S1 Fig Panel A and S1 Fig Panel B | Advion |
| 989 | Advion SCG, N=29 vs IE, N=29 | S1 Fig Panel A and S1 Fig Panel B | Advion |
| 991 | Advion SCG, N=29 vs IE, N=29 | S1 Fig Panel A and S1 Fig Panel B | Advion |
| 994 | Advion SCG, N=29 vs IE, N=29 | S1 Fig Panel A and S1 Fig Panel B | Advion |
| 999 | Advion SCG, N=29 vs IE, N=29 | S1 Fig Panel A and S1 Fig Panel B | Advion |
| 1016 | Advion SCG, N=29 vs IE, N=29 | S1 Fig Panel A and S1 Fig Panel B | Advion |
| 1026 | Advion SCG, N=29 vs IE, N=29 | S1 Fig Panel A and S1 Fig Panel B | Advion |
| 1035 | Advion SCG, N=29 vs IE, N=29 | S1 Fig Panel A and S1 Fig Panel B | Advion |
| 1036 | Advion SCG, N=29 vs IE, N=29 | S1 Fig Panel A and S1 Fig Panel B | Advion |
| 1051 | Advion SCG, N=29 vs IE, N=29 | S1 Fig Panel A and S1 Fig Panel B | Advion |
| 1057 | Advion SCG, N=29 vs IE, N=29 | S1 Fig Panel A and S1 Fig Panel B | Advion |
| 1064 | Advion SCG, N=29 vs IE, N=29 | S1 Fig Panel A and S1 Fig Panel B | Advion |
| 1089 | Advion SCG, N=29 vs IE, N=29 | S1 Fig Panel A and S1 Fig Panel B | Advion |
| 1146 | Advion SCG, N=29 vs IE, N=29 | S1 Fig Panel A and S1 Fig Panel B | Advion |
| 1148 | Advion SCG, N=29 vs IE, N=29 | S1 Fig Panel A and S1 Fig Panel B | Advion |
| 1157 | Advion SCG, N=29 vs IE, N=29 | S1 Fig Panel A and S1 Fig Panel B | Advion |
| 1167 | Advion SCG, N=29 vs IE, N=29 | S1 Fig Panel A and S1 Fig Panel B | Advion |
| 419 | Advion Training sets (N=25) and Blind samples (N=4) SCG vs IE | S1 Fig Panel C and S1 Fig Panel D | Advion |
| 448 | Advion Training sets (N=25) and Blind samples (N=4) SCG vs IE | S1 Fig Panel C and S1 Fig Panel D | Advion |
| 473 | Advion Training sets (N=25) and Blind samples (N=4) SCG vs IE | S1 Fig Panel C and S1 Fig Panel D | Advion |
| 513 | Advion Training sets (N=25) and Blind samples (N=4) SCG vs IE | S1 Fig Panel C and S1 Fig Panel D | Advion |
| 541 | Advion Training sets (N=25) and Blind samples (N=4) SCG vs IE | S1 Fig Panel C and S1 Fig Panel D | Advion |
| 546 | Advion Training sets (N=25) and Blind samples (N=4) SCG vs IE | S1 Fig Panel C and S1 Fig Panel D | Advion |
| 552 | Advion Training sets (N=25) and Blind samples (N=4) SCG vs IE | S1 Fig Panel C and S1 Fig Panel D | Advion |
| 561 | Advion Training sets (N=25) and Blind samples (N=4) SCG vs IE | S1 Fig Panel C and S1 Fig Panel D | Advion |
| 567 | Advion Training sets (N=25) and Blind samples (N=4) SCG vs IE | S1 Fig Panel C and S1 Fig Panel D | Advion |
| 695 | Advion Training sets (N=25) and Blind samples (N=4) SCG vs IE | S1 Fig Panel C and S1 Fig Panel D | Advion |
| 699 | Advion Training sets (N=25) and Blind samples (N=4) SCG vs IE | S1 Fig Panel C and S1 Fig Panel D | Advion |
| 732 | Advion Training sets (N=25) and Blind samples (N=4) SCG vs IE | S1 Fig Panel C and S1 Fig Panel D | Advion |
| 753 | Advion Training sets (N=25) and Blind samples (N=4) SCG vs IE | S1 Fig Panel C and S1 Fig Panel D | Advion |
| 771 | Advion Training sets (N=25) and Blind samples (N=4) SCG vs IE | S1 Fig Panel C and S1 Fig Panel D | Advion |
| 773 | Advion Training sets (N=25) and Blind samples (N=4) SCG vs IE | S1 Fig Panel C and S1 Fig Panel D | Advion |
| 926 | Advion Training sets (N=25) and Blind samples (N=4) SCG vs IE | S1 Fig Panel C and S1 Fig Panel D | Advion |
| 941 | Advion Training sets (N=25) and Blind samples (N=4) SCG vs IE | S1 Fig Panel C and S1 Fig Panel D | Advion |
| 951 | Advion Training sets (N=25) and Blind samples (N=4) SCG vs IE | S1 Fig Panel C and S1 Fig Panel D | Advion |
| 958 | Advion Training sets (N=25) and Blind samples (N=4) SCG vs IE | S1 Fig Panel C and S1 Fig Panel D | Advion |
| 976 | Advion Training sets (N=25) and Blind samples (N=4) SCG vs IE | S1 Fig Panel C and S1 Fig Panel D | Advion |
| 989 | Advion Training sets (N=25) and Blind samples (N=4) SCG vs IE | S1 Fig Panel C and S1 Fig Panel D | Advion |
| 991 | Advion Training sets (N=25) and Blind samples (N=4) SCG vs IE | S1 Fig Panel C and S1 Fig Panel D | Advion |
| 994 | Advion Training sets (N=25) and Blind samples (N=4) SCG vs IE | S1 Fig Panel C and S1 Fig Panel D | Advion |
| 1026 | Advion Training sets (N=25) and Blind samples (N=4) SCG vs IE | S1 Fig Panel C and S1 Fig Panel D | Advion |
| 1034 | Advion Training sets (N=25) and Blind samples (N=4) SCG vs IE | S1 Fig Panel C and S1 Fig Panel D | Advion |
| 1035 | Advion Training sets (N=25) and Blind samples (N=4) SCG vs IE | S1 Fig Panel C and S1 Fig Panel D | Advion |
| 1057 | Advion Training sets (N=25) and Blind samples (N=4) SCG vs IE | S1 Fig Panel C and S1 Fig Panel D | Advion |
| 1089 | Advion Training sets (N=25) and Blind samples (N=4) SCG vs IE | S1 Fig Panel C and S1 Fig Panel D | Advion |
| 1091 | Advion Training sets (N=25) and Blind samples (N=4) SCG vs IE | S1 Fig Panel C and S1 Fig Panel D | Advion |
| 1108 | Advion Training sets (N=25) and Blind samples (N=4) SCG vs IE | S1 Fig Panel C and S1 Fig Panel D | Advion |
| 1127 | Advion Training sets (N=25) and Blind samples (N=4) SCG vs IE | S1 Fig Panel C and S1 Fig Panel D | Advion |
| 1146 | Advion Training sets (N=25) and Blind samples (N=4) SCG vs IE | S1 Fig Panel C and S1 Fig Panel D | Advion |
| 1148 | Advion Training sets (N=25) and Blind samples (N=4) SCG vs IE | S1 Fig Panel C and S1 Fig Panel D | Advion |
| 1154 | Advion Training sets (N=25) and Blind samples (N=4) SCG vs IE | S1 Fig Panel C and S1 Fig Panel D | Advion |
| 1160 | Advion Training sets (N=25) and Blind samples (N=4) SCG vs IE | S1 Fig Panel C and S1 Fig Panel D | Advion |
| 1167 | Advion Training sets (N=25) and Blind samples (N=4) SCG vs IE | S1 Fig Panel C and S1 Fig Panel D | Advion |
| 1168 | Advion Training sets (N=25) and Blind samples (N=4) SCG vs IE | S1 Fig Panel C and S1 Fig Panel D | Advion |
| 1169 | Advion Training sets (N=25) and Blind samples (N=4) SCG vs IE | S1 Fig Panel C and S1 Fig Panel D | Advion |
| 1191 | Advion Training sets (N=25) and Blind samples (N=4) SCG vs IE | S1 Fig Panel C and S1 Fig Panel D | Advion |
| 1194 | Advion Training sets (N=25) and Blind samples (N=4) SCG vs IE | S1 Fig Panel C and S1 Fig Panel D | Advion |
