# Supplementary material for: Distinguishing patients with idiopathic epilepsy from solitary cysticercus granuloma epilepsy and biochemical phenotype assessment using a serum biomolecule profiling platform
Source: PLoS One. 2020 Aug 21;15(8):e0237064. doi: 10.1371/journal.pone.0237064 (PMC7527271; doi:10.1371/journal.pone.0237064)
Supplement: S12 File — (DOCX) [file pone.0237064.s018.docx]

**S12_File_Remarks Concerning Methods:**

**Calculation of Ratio values for IPA analysis.**

The number of sera presented in the tables is defined by the number of individual patient serum samples observed with each presented protein. The number of “Hits” is the number of individual scans associated with each protein identification from “Proteome Discoverer” for all sera tested. For example, the MEGF protein in the “SCG vs IE” table listed as “MEGF 6 (63): 2 (12)” would indicate the protein was observed in 6 SCG sera with 63 “Hits” and in 2 IE sera with 12 “Hits”.

The “Hit” ratio utilized in IPA for expression would be calculated using the following Excel equation:

=imlog2(sum(((“#Hits_SCG”+1)/(“#Hits_IE”+1))).

An exact paste text representation would be: “=imlog2((((63+1)/(12+1)))”.

It should be noted, we have used the “+1” in the equation to prevent division by zero and answers of infinity in a conservative approach.

**Concerning PCV selection Calculations:**

The PCV determination rules state a peak area value greater than the PCV is required for a peak to be classified with the group with the higher mean value. This brings into question the issue of peaks of equal value to the PCV. By requiring a value to be greater than the PCV, the authors have sided with a more conservative approach requiring the value to be more than just equal to the PCV
